# Robust polyfunctional CD8+ and CD4+ T cell responses in HLA-A*0201/DR1 transgenic mice following vaccination with modified vaccinia virus Ankara-based vaccines delivering Lassa virus glycoprotein or nucleoprotein

**DOI:** 10.1099/jgv.0.002142

**Published:** 2025-09-01

**Authors:** Alina Tscherne, Georgia Kalodimou, Sylvia Jany, Astrid Freudenstein, Satendra Kumar, Veronika Pilchová, Theresa Friebis, Gabriel Maiwald, Isabella Panhofer, Gerd Sutter†, Asisa Volz

**Affiliations:** 1 Division of Virology, Department of Veterinary Sciences, Ludwig Maximilians University (LMU Munich), 85764 Oberschleißheim, Germany; 2 German Centre for Infection Research, Partner Site Munich, 85764 Oberschleißheim, Germany; 3 Institute of Virology, University of Veterinary Medicine Hannover, 30559 Hannover, Germany; 4 German Centre for Infection Research, Partner Site Hannover-Braunschweig, 30559 Hannover, Germany

**Keywords:** emerging viruses, glycoprotein, modified vaccinia virus Ankara, Lassa virus, nucleoprotein, T cell vaccine, viral vector vaccine

## Abstract

Lassa virus (LASV) is circulating in rodents in several countries in West Africa and is the causative agent of the zoonotic disease Lassa fever. Several vaccine candidates have been successfully tested in preclinical and clinical research, while no LASV-specific vaccines or antiviral treatments have been licensed to date. Approximately 500,000 human cases of Lassa fever are estimated to occur every year. However, the high percentage (~80%) of asymptomatic cases and the low frequency of reporting systems in endemic regions demonstrate that Lassa fever cases are highly underreported. Given the frequent spread of the virus by travellers to non-endemic regions, the need for effective vaccines and treatments becomes clear. Here, we describe the generation and preclinical evaluation of two recombinant Lassa virus candidate vaccines, MVA-GP and MVA-NP, which are based on the highly attenuated modified vaccinia virus Ankara (MVA) strain. Constructed in the MVA vector, the MVA-GP vaccine delivers the glycoprotein (GP) of the prototype LASV Josiah strain (lineage IV), whereas the MVA-NP vaccine expresses the nucleoprotein (NP) from the Lassa virus Togo strain (lineage VII). Two immunizations of either MVA-GP or MVA-NP induced substantial polyfunctional Lassa virus-specific CD8^+^ and CD4^+^ T cell responses, respectively, in humanized *HLA-A2.1-/HLA-DR1-transgenic H-2 class I-/class II-knockout* mice (HLA-A*0201/DR1 transgenic mice). The identified human Lassa virus-specific T cell epitopes were in agreement with recently discovered T cell epitopes found in Lassa fever survivors. Further studies are warranted to characterize these recombinant MVA-Lassa virus vaccine candidates in other preclinical models and investigate their potential to be characterized in clinical studies in humans.

## Introduction

Lassa virus (LASV), causative agent of the zoonotic disease Lassa fever (LF), is endemic in several countries in West Africa [1,4]. Four major lineages (I–IV) have been identified for LASV, with an additional four proposed lineages (V–VIII) being recognized based on phylogenetic analysis over the last years [5,6]. Lineages I, II, III and VI are circulating in different areas in Nigeria [5,7], whereas lineage IV is circulating in Liberia, Guinea and Sierra Leone [6,7]. Lineage V, most likely a sub-lineage from lineage IV, was found in Mali and Côte d'Ivoire [3,7, 8]. Lineages VII and VIII were detected in Togo and Benin, respectively [9,10]. Interestingly, all lineages show inter-lineage genetic diversity [5,9, 11], thus hampering the development of effective vaccines and antivirals, as medical countermeasures against one lineage might be less effective against other lineages. The glycoprotein gene sequence used in this study is based on the LASV Josiah strain (lineage IV), the prototype strain for LASV research. The nucleoprotein gene sequence used in this study is based on the LASV Togo strain (lineage VII), which was associated with the first autochthonous LF case in Germany in 2016 [9].

LASV is an enveloped, negative-sense, and single-stranded RNA virus, which belongs to the *Arenaviridae* family, genus *Mammarenavirus*, and is classified as Old World mammarenavirus complex, closely related to Mopeia virus and Lujo virus [12]. LASV virions contain a bi-segmented and ambisense RNA genome of about 10 kb in size [13]. Each segment consists of two non-overlapping ORFs of opposite polarity separated by a non-coding region forming a hairpin structure [7]. The large segment encodes a ~11 kDa zinc-binding matrix protein (Z) [14] and a ~200 kDa RNA-dependent RNA polymerase (L) [13,15]. The small segment encodes a ~63 kDa nucleoprotein (NP) and a ~75 kDa glycoprotein precursor (GPC), which is post-translationally cleaved into stable signal peptide (SSP), GP1 and GP2 [16,17], and is the only protein located at the viral surface. Upon infection, the GP1 subunit binds to the host cell receptor (*α*-dystroglycan), allowing the virus to be internalized via endocytosis and transported to the late endosome, where GP1 dissociates. GP2 subunit gets exposed and mediates membrane fusion to release the viral genome into the cytoplasm of infected cells. NP plays an essential role in different stages of the viral life cycle, such as encapsidation of RNA, transcription and translation of viral RNA together with the L protein, and inhibiting the host immune response (e.g. interferon pathway) [18,19].

The main reservoir of LASV is *Mastomys natalensis* [20], a multimammate rat species, in which LASV causes an inapparent and age-dependent transient or persistent infection [21,22]. The virus is shed via rodent ex- and secreta, including urine, faeces or saliva. Infections of humans with LASV predominantly occur either by ingestion of contaminated food or via direct contact with infected rats [23,24]. It is estimated that 300,000–500,000 humans are infected every year and ~5,000 people die from LF [25]. Given the high number of asymptomatic LF cases and low frequency of standardized surveillance and reporting programmes in endemic regions, the actual number of reported infections is highly underreported [26]. Most human infections with LASV occur via rodent-to-human transmission; however, human-to-human transmission, mainly within domiciliary groups and among healthcare workers due to inadequate infection control measures, is reported occasionally [24,27, 28]. The vast majority of LASV infections (~80%) are asymptomatic, cause mild symptoms or even remain undetected. However, a moderate to severe form of LF associated with organ failure and haemorrhagic manifestations is observed in ~20% of LASV-infected individuals [23]. Severe complications are mainly observed in immunocompromised individuals, children under the age of 5 and pregnant women [29,32].

Although several vaccines have been tested successfully in preclinical and clinical trials, no vaccines or preventive treatments have received approval yet. Given the lack of effective antivirals and vaccines, and the high pathogenicity of and variability between LASV strains, the World Health Organization and the Coalition for Epidemic Preparedness Innovations have categorized the development of effective LASV vaccines as a high priority [33,35]. Different vaccine platforms, such as vesicular stomatitis virus (VSV) vectored [36,38], vaccinia virus (VACV) vectored [39,40], modified vaccinia virus Ankara (MVA) vectored [41,43], measles virus (MV) vectored [44,45] and chimpanzee adenovirus vectored [46], as well as DNA-based [47,50], mRNA-based [51,52] and live-attenuated [53] vaccines, targeting GP and/or NP, have been used in preclinical studies. Viral vectored vaccines showed protective efficacy in different animal models (small rodents to non-human primates) and the induction of antigen-specific IgG antibody titres and/or LASV-specific CD4/CD8 T cells (for review, see [54]). Over the last years, the most promising candidates (e.g. VSVΔG/LASVGPC and MV-LASV) have entered evaluation in phase I/II clinical trials, mainly focusing on tolerability and immunogenicity (humoral and cellular immunity) (e.g. NCT04794218, NCT04055454, NCT05868733 and NCT06546709) (for review, see [54]). MV-LASV, expressing LASV GP and NP, has been tested in a phase I clinical trial and induced antigen-specific IgG binding antibodies and LASV-specific T cells after two immunizations, but failed to induce neutralizing antibody responses [55]. Since some of the clinical studies evaluating LASV vaccines based on different vaccine platforms are still ongoing or finished just recently, only limited information on the obtained study results is publicly available.

The correlate of protection against Lassa virus is not yet defined; however, published data from animal models and human LF survivors suggest that the activation of a robust T cell response against nucleoprotein and/or glycoprotein might be sufficient to provide a protective immunity against LASV [56]. Furthermore, in contrast to other haemorrhagic fever viruses, such as Ebola virus (EBOV) [57], the induction of high antibody responses did not correlate with disease outcome in humans [56,58], and LASV-specific T cells are observed in survivors for months and even years after recovery [59,61].

MVA, licensed as a vaccine against human smallpox and more recently also against human mpox, has been used for decades as a safe viral vector to deliver antigens from different pathogens (for review, [62]). Due to its strict attenuation by passaging its ancestor VACV more than 500 times on chicken embryonic fibroblasts (CEF), MVA no longer replicates in humans and is cleared from the human body within a few days after immunization [63]. In various preclinical and clinical trials, the suitability of MVA to induce strong cellular and humoral immune responses has been confirmed (for review, see [62]). Kennedy *et al.* [41] developed a recombinant MVA-based LASV vaccine expressing NP (MVA-LassNP) from the prototype Josiah strain (lineage IV) and confirmed its immunogenicity and protective efficacy in a guinea pig challenge model. Furthermore, Salvato *et al.* [42] generated an MVA-based LASV vaccine expressing both GP and Z (designated as GEO-LM01), which forms virus-like particles (VLPs). The VLP vaccine protected CBA/J mice against a lethal challenge infection with a Mopeia–Lassa reassortant virus. Both MVA-LassNP and GEO-LM01 demonstrated the potential of using MVA to develop efficacious vaccine candidates against LF.

In this study, we aimed to evaluate the capability of two MVA-based vaccines against LASV to induce robust T cell responses. The MVA-GP vaccine candidate expressed the LASV-GP from the LASV Josiah strain (lineage IV), and the MVA-NP vaccine candidate expressed the LASV-NP from the LASV Togo strain (lineage VII). Activation of LASV-specific cellular immunity was evaluated in humanized *HLA-A2.1-/HLA-DR1-transgenic H-2 class I-/class II-knockout* mice (HLA-A*0201/DR1 transgenic mice). Prime-boost vaccination of HLA-A*0201/DR1 transgenic mice induced a robust activation of LASV-epitope-specific T cells also found in LF survivors [64,65]. These results will be advantageous to further contribute to the development of LASV preventative and therapeutic approaches for humans.

## Methods

### Cell cultures

CEF were isolated from 11-day-old specific pathogen-free (SPF) chicken embryos (VALO, Cuxhaven, Germany) following established protocols as published recently [66]. Briefly, the eggshells of embryonated eggs were carefully opened with scissors, and the embryos were lifted out and transferred to Petri dishes containing sterile PBS (Thermo Fisher Scientific, Planegg, Germany). Head, legs, organs and wings were removed, and the remaining torso was homogenized by pressing it through a syringe. In addition, the homogenized tissues were treated with 0.1% trypsin-EDTA (Thermo Fisher Scientific, Planegg, Germany), and the remaining cell suspension was filtered to remove embryo clumps. Subsequently, the cell suspension was centrifuged, and the pellet was resuspended in VP-SFM medium (Thermo Fisher Scientific, Planegg, Germany), supplemented with 10% heat-inactivated FBS (Thermo Fisher Scientific, Planegg, Germany) and 1% l-glutamine (Thermo Fisher Scientific, Planegg, Germany). CEF cells were transferred to T175 cell culture flasks and incubated overnight at 37 °C. HaCat cells (CLS Cell Lines Service GmbH, Eppelheim, Germany) were cultured in Minimum Essential Medium Eagle (MEM) (Sigma-Aldrich, Taufkirchen, Germany), supplemented with 7% FBS and 1% MEM non-essential amino acid solution (Sigma-Aldrich, Taufkirchen, Germany). Vero E6 cells (ATCC CCL-81, Manassas, VA, USA) were cultured in Dulbecco’s Modified Eagle’s Medium (Sigma-Aldrich, Taufkirchen, Germany), supplemented with 10% FBS and 1% MEM non-essential amino acid solution. All cells were kept at 37 °C with 5% CO_2_. For infection experiments, the percentage of FBS in the culture medium was reduced to 2%.

### Plasmid construction and generation of recombinant MVA vaccine candidates

MVA transfer plasmids pIIIH5red-LASV-GP (pIII-GP) and pLW73-LASV-NP (pLW73-NP) were used to generate the candidate vaccines MVA-LASV-GP (MVA-GP) and MVA-LASV-NP (MVA-NP), respectively, following the established protocols for rapid development of recombinant MVAs suitable for clinical use as published recently [66,67]. As target antigens, coding sequences of the full-length glycoprotein (GP) from the LASV Josiah strain (lineage IV, GenBank ID: J04324.1) and nucleoprotein (NP) from the LASV Togo strain (lineage VII, GenBank ID: KU961971.1) [9] were obtained from the PubMed National Center for Biotechnology Information (NCBI) webpage and modified, including the addition of restriction enzymes (BamHI/NotI for LASV-GP; BamHI/XbaI for LASV-NP) at the 5′- and 3′-end and codon optimization for unimpaired protein expression by the MVA vector. For the latter, silent mutations were introduced at TTTTTNT regions to remove termination signals for vaccinia virus transcription. The modified gene sequences were synthesized by gene synthesis (Genewiz, Leipzig, Germany) and cloned into the MVA transfer plasmids pIIIH5red and pLW73 [68], respectively. To obtain MVA-GP, monolayers of 90–95% confluent CEF cells were infected with MVA-GFP at a multiplicity of infection (MOI) of 1 and incubated for 40 min at 37 °C. Subsequently, the inoculum was replaced by VP-SFM medium (+2% FBS, 1% l-glutamine), and cells were transfected with 1 µg of pIII-GP, using X-tremeGENE HP DNA Transfection Reagent (Sigma-Aldrich, Taufkirchen, Germany) according to the manufacturer’s instructions. To obtain MVA-NP, monolayers of 90–95% confluent CEF cells were infected with MVA-mCherry at an MOI of 1 and incubated for 40 min at 37 °C. Subsequently, the inoculum was replaced by VP-SFM medium (+2% FBS, 1% l-glutamine), and cells were transfected with 1 µg of pLW73-NP, using X-tremeGENE HP DNA Transfection Reagent (Sigma-Aldrich, Taufkirchen, Germany) according to the manufacturer’s instructions. Forty-eight hours after incubation, cell cultures were collected, freeze-thawed, serially diluted and used to infect monolayers of 90–95% confluent CEF cells. Recombinant MVA-GP and MVA-NP were clonally isolated by consecutive rounds of plaque isolation using the co-expression of the fluorescent marker proteins mCherry and GFP, respectively. To obtain high-titre virus preparations, recombinant MVA-GP and MVA-NP were amplified in CEF cells, purified by centrifugation through sucrose cushion and re-suspended in Tris-HCl buffer (pH 9.0).

### *In vitro* characterization of recombinant MVA-LASV-GP and MVA-LASV-NP

MVA-GP and MVA-NP were tested for genetic stability by PCR and for viral growth by multiple-step growth experiments following protocols as published recently [66]. To confirm the genetic stability of the recombinant MVA viruses, monolayers of 90–95% confluent CEF cells were infected with MVA-GP or MVA-NP at an MOI of 1 and incubated for 24 h. Subsequently, viral DNA was extracted using the NucleoSpin Blood QuickPure (Macherey-Nagel, Düren, Germany) according to the manufacturer’s instructions. PCR analysis was done by using Taq DNA polymerase (Thermo Fisher Scientific, Planegg, Germany) and oligonucleotide sequences specific for the six major deletion sites (Del I–VI) and the *C7L* gene locus [66] (MVA_Del1-F: CTTTCGCAGCATAAGTAGTATGTC, MVA_Del1-R: CATTACCGCTTCATTCTTATATTC; MVA_Del2-F: GGGTAAAATTGTAGCATCATATACC MVA_Del2-R: AAAGCTTTCTCTCTAGCAAAGATG; MVA_Del3-F: GATGAGTGTAGATGCTGTTATTTTG; MVA_Del3-R: GCAGCTAAAAGAATAATGGAATTG; MVA_Del4-F: AGATAGTGGAAGATACAACTGTTACG; MVA_Del4-R: TCTCTATCGGTGAGATACAAATACC; MVA_Del5-F: CGTGTATAACATCTTTGATAGAATCAG; MVA_Del5-R: AACATAGCGGTGTACTAATTGATTT, MVA_Del6-F: CTACAGGTTCTGGTTCTTTATCCT; MVA_Del6-R: CACGGTCAATTAACTATAGCTCTG; C7L-F: CATGGACTCATAATCTCTATAC, C7L-R: ATGGGTATACAGCACGAATTC). Correct insertion of LASV-NP into the intergenic region between the two MVA genes, *MVA-069R* and *MVA-070L*, was confirmed using the primers MVA-069R/070 L-5′: ATTCTCGCTGAGGAGTTGG and MVA-069R/070 L-3′: GTCGTGTCTACAAAAGGAG [66]. The genetic identity of LASV-GP and LASV-NP was confirmed by using primers targeting the inserted gene sequences (LASV-GP-for: CCCTGCCTCTCCATATACTC; LASV-GP-rev: ACTTTCCACTTGTCCATCCC; LASV-NP-for: TTACTAACACTTGCTGCTGAC; LASV-NP-rev: CCTCCCTTCTTTTTCTTCTC). Viral growth was determined by a multiple-step growth analysis with subsequent counting of p.f.u. following established methods [66]. Briefly, monolayers of 90–95% confluent CEF or HaCat cells were infected with recombinant MVA-GP, recombinant MVA-NP or non-recombinant MVA (MVA) at an MOI of 0.05 and cells were incubated for 30 min at 4 °C. Subsequently, the inoculum was replaced by growth medium, and cells were incubated at 37 °C. After 0, 4, 8, 12, 24, 48 and 72 h post-infection (hpi), cell cultures were collected and frozen at −20 °C. Cell cultures were thawed, serially diluted and used to infect monolayers of 90–95% confluent CEF cells. The infected cells were incubated for 48 h at 37 °C and subsequently fixed with ice-cold methanol/acetone (1:1). Cells were incubated with a primary anti-VACV-specific antibody (1:2,000; OriGene Technologies GmbH, Herford, Germany) for 1 h at room temperature. Subsequently, cells were incubated with a peroxidase-labelled secondary antibody. Positively stained plaques were visualized by adding KPL TrueBlue™ Peroxidase Substrate (HiSS Diagnostics GmbH, Freiburg im Breisgau, Germany). Plaques were counted, and the results were expressed as p.f.u. ml^−1^.

### Western blot analysis of recombinant proteins

CEF cells were infected with recombinant MVA-GP, MVA-NP or non-recombinant MVA at MOI 10 or remained uninfected (Mock). Infected cells were harvested at time-points between 0 and 24 hpi to prepare cell lysates. Cells were scraped, transferred to microcentrifuge tubes and centrifuged at top speed. Subsequently, cells were lysed by resuspending the pellet in ice-cold Triton X lysis buffer (1% Triton X-100, 25 mM Tris and 1 M NaCl), incubated for 30 min at 4 °C and centrifuged at top speed. Supernatants containing the lysed proteins were collected and analysed by SDS-PAGE and Western blotting. Protein lysates were mixed with 4 × reducing agent containing *β*-mercaptoethanol (Bio-Rad, Munich, Germany) and boiled at 95 °C for 5 min. Subsequently, samples were loaded on 4–15% Mini-PROTEAN® TGX™ Precast Protein Gels (Bio-Rad, Munich, Germany) to separate the proteins in the lysates, which were subsequently transferred onto nitrocellulose membranes. Recombinant LASV-NP or LASV-GP was detected by incubating the membranes overnight with primary antibodies targeting LASV-NP (1:1,000; Alpha Diagnostics, San Antonio, TX, USA) or LASV-GP (1:1,000; Abcam, Cambridge, UK). Anti-mouse (1:5,000; Agilent Dako, Glostrup, Denmark) or anti-rabbit IgG antibodies (1:5,000; Cell Signaling Technology, Danvers, MA, USA), conjugated to HRP, were used as secondary antibodies. To visualize the target proteins, membranes were incubated with SuperSignal West Dura Extended Duration Substrate (Thermo Fisher Scientific, Planegg, Germany) and analysed by the ChemiDocTMMP Imaging System (Bio-Rad, Munich, Germany).

### Immunofluorescence staining of recombinant proteins

Vero E6 cells grown on coverslips (Marienfeld Superior, Lauda-Königshofen, Germany) were infected with recombinant MVA-GP or MVA-NP or non-recombinant MVA (MVA) at an MOI of 0.1 or remained uninfected (Mock) and incubated for 24 h at 37 °C. The conditions for immunofluorescence staining (IF) were published recently [66,69]. Briefly, cells were fixed with 4% paraformaldehyde/PBS for 10 min on ice and permeabilized with 0.1% Triton X-100/PBS. To detect LASV-GP or LASV-NP, primary antibodies targeting LASV-GP (1:5,000; Abcam, Cambridge, UK) or LASV-NP (1:5,000; Cambridge Biologics, MA, USA) were used. A polyclonal goat anti-rabbit IgG (H+L) highly cross-adsorbed secondary antibody, Alexa Fluor™ 488 (1:1,000; Thermo Fisher Scientific, Planegg, Germany), was used to visualize GP- or NP-specific staining by green fluorescence staining. Cell nuclei were counterstained with 1 µg ml^−1^ of DAPI (Sigma-Aldrich, Taufkirchen, Germany). Cells were analysed using the Keyence BZ-X700 microscope (Keyence, Neu-Isenburg, Germany) with a Plan Apochromat 100 × objective.

**Ethical approval**. *HLA-A2.1-/HLA-DR1-transgenic H-2 class I-/class II*-knockout mice (HLA-A*0201/DR1 transgenic mice) were obtained from the Institute Pasteur/Charles River Laboratories and bred in the biosafety level (BSL)−2 animal facility of the Institute for Infectious Diseases and Zoonoses, LMU Munich, Germany, under SPF conditions. All animal experiments were handled and conducted in compliance with the European and national regulations for animal experimentation (European Directive 2010/63/EU; Animal Welfare Acts in Germany) and the Animal Welfare Act, approved by the Regierung von Oberbayern, Bavaria, Germany (protocol code ROB-55.2–2532.Vet_02-22-71). All animals were kept at a 12/12 dark/light cycle, humidity of 45–65% and a temperature of 20–24 °C and had access to food and water *ad libitum*.

### Vaccination experiments in mice

Two different mouse studies were performed to evaluate the immunogenicity of recombinant MVA-GP and MVA-NP. For both experiments, mice immunized with the non-recombinant MVA vector (MVA) served as negative controls. The study design included the use of both sexes, which were mixed across the experimental groups. For the characterization of MVA-GP, a total of 15 HLA-A*0201/DR1 transgenic mice were divided into two groups (*n*=10 for MVA-GP vaccine group; *n*=5 for the MVA control group) and immunized twice over a 3-week interval with 10^7^ p.f.u. of recombinant MVA-GP or MVA into the left hind leg via the intramuscular (IM) route under isoflurane anaesthesia. A dose of 10^7^ p.f.u. per application has proven safe and immunogenic in this mouse strain in our previous immunization studies and was, therefore, used for this study [67,70, 71]. For the characterization of MVA-NP, a total of 15 HLA-A*0201/DR1 transgenic mice were divided into two groups (*n*=10 for MVA-NP vaccine group; *n*=5 for the MVA control group) immunized twice over a 3-week interval with 10^7^ p.f.u. of recombinant MVA-NP or MVA into the left hind leg via the IM route under isoflurane anaesthesia. Following immunization, mice were weighed daily and monitored for clinical abnormalities (e.g. behaviour) and general conditions (e.g. weight loss or piloerection), which had been predefined and approved in a supervision protocol. Mice were euthanized by cervical dislocation under deep isoflurane anaesthesia due to experimental settings on day 35 post-first immunization. At necropsy, spleens were collected for subsequent analysis by ELISpot and IFN-*γ*/TNF-*α* ICS FACS analysis.

### Peptides used for stimulation (generation of peptides, design of peptide pools and peptide prediction)

The protein sequences of LASV-GP (GenBank ID: AAA46286.1) and LASV-NP (GenBank ID: AMR44578.1) were used for predicting MHC binding peptides *in silico*. For LASV-GP, a mixture of published and predicted peptides was used for the experiments (Table S1, available in the online Supplementary Material). For LASV-NP, predicted/published peptides and 15-mer peptides with an 11-mer overlap that spanned the entire sequence (Tables S1 and S2) were used. For the peptide prediction, we identified peptides that bound to the human MHC class I (MHC-I) allele HLA-A*02:01 for LASV-GP and LASV-NP. The prediction of MHC-I binding peptides (8–11 mer) was done using the Immune Epitope Database (IEDB) tools MHC-I Binding Predictions and MHC-I Processing Predictions [72,73], using the NetMHCpan 4.1 prediction method [74]. To narrow down the prediction results list, we selected a median binding percentile cutoff of 5 and an IC_50_ cutoff of 1,000 nM. From this list of peptides, we selected those that had been previously identified in LF patients plus the top 2–3 peptides according to the prediction results. We also predicted LASV-GP and LASV-NP MHC class II (MHC-II) binding peptides (15 mer) for the human allele HLA-DRB1*01:01 with the IEDB tool MHC-II Binding Predictions [72,73], using the NetMHCIIpan 4.0 prediction method [74]. We screened the results for peptides with a median binding percentile of 10 or less and an IC50 of 2,000 or less. We identified MHC-II binding peptides that had been previously identified in LF patients and selected an additional 1–2 peptides. LASV-GP-specific peptides were tested individually or pooled into pools of two peptides depending on whether they were MHC-I or MHC-II binding peptides (Table S1). LASV-NP 15-mer overlapping peptides (Table S2) were organized into pools using a peptide matrix strategy described previously [75]. In addition, LASV-NP-specific peptides were tested individually or pooled into a pool of two peptides based on their prediction results (Table S1). The top three MHC-I and MHC-II binding peptides were tested individually, and the remainder were combined into a pool. VACV-specific responses were tested using the immunodominant CD8 T cell epitope A6(L)_6-14_ (VLYDEFVTI) (Table S1) [76,77]. Peptides were synthesized by Thermo Fisher Scientific and dissolved to a concentration of 2 mg ml^−1^ in DMSO (Sigma-Aldrich, Taufkirchen, Germany) under sterile conditions. Reconstituted peptides were aliquoted and stored at −20 °C.

### T cell analysis by enzyme-linked immunospot

The enzyme-linked immunospot (ELISpot) assay was performed to analyse IFN-*γ*-producing T cells in the spleens of immunized mice *ex vivo* (*n*=6–10 for MVA-GP or MVA-NP vaccine groups; *n*=4–5 for MVA control group; the exact numbers are specified in the figure legends for each experiment). On day 35 after the first vaccination, mice were euthanized, and splenocytes were isolated as described previously [66]. In brief, spleens were passed through a cell strainer and incubated with Red Blood Cell Lysis Buffer (Sigma-Aldrich, Taufkirchen, Germany). Cells were centrifuged, washed with sterile PBS (Thermo Fisher Scientific, Planegg, Germany) and resuspended in RPMI medium (+10% FBS, 1% HEPES and 1% penicillin/streptomycin). The ELISpot assay was conducted following the manufacturer’s instructions (ELISpot Plus Mouse IFN-*γ* (ALP), Mabtech AB, Nacka Strand, Sweden). Briefly, 2×10^5^ splenocytes well^−1^ were transferred to 96-well cell culture plates and stimulated with individual peptides (2 µg ml^−1^) or pools of predicted, published or overlapping peptides (2 µg ml^−1^ per peptide) (Thermo Fisher Scientific, Planegg, Germany (Tables S1 and S2) as indicated. Non-stimulated cells and cells stimulated with phorbol-12-myristate-13-acetate (PMA)/ionomycin served as negative and positive controls, respectively. Subsequently, cells were incubated for 48 h at 37 °C and stained following the manufacturer’s instructions. Single spots were counted and analysed by an automated ELISpot plate reader (A.EL.VIS Eli.Scan, A.EL.VIS ELISpot Analysis Software, Hannover, Germany). Results are shown as spot-forming cells (SFC) per 10^6^ cells, after the subtraction of mock-stimulated cells for each animal.

### T cell analysis by intracellular cytokine staining

On day 35 after the first vaccination, mice were euthanized, and splenocytes were isolated as described above (*n*=6–8 for MVA-GP or MVA-NP vaccine group; *n*=4 for MVA control group; the exact numbers are specified in the figure legends for each experiment). The conditions for IFN-*γ*/TNF-*α* intracellular cytokine staining (ICS) have been published recently [66]. Briefly, 1×10^6^ splenocytes well^−1^ were transferred to 96-well round-bottom plates and stimulated with pools of predicted and overlapping or individual peptides (8 µg ml^−1^ per peptide) (Tables S1 and S2) for 2 h at 37 °C. Brefeldin A (Biolegend, San Diego, CA, USA) was added, and the cells were stimulated for another 4 h at 37 °C. Non-stimulated cells and cells stimulated with PMA/ionomycin served as negative and positive controls, respectively. After incubation, cells were stained extracellularly using anti-mouse CD3 PE/Cy7, anti-mouse CD4 Brilliant Violet 421™, anti-mouse CD8*α* Alexa Fluor® 488 and anti-mouse CD16/32 (Table S3). The fixable viability dye Zombie Aqua (1:1,000; Biolegend, San Diego, CA, USA) was used for staining dead cells. Next, cells were fixed with fixation buffer and permeabilized with permeabilization wash buffer according to the manufacturer’s protocol (both by Biolegend, San Diego, CA, USA), and cells were stained intracellularly using anti-mouse IFN-*γ* and anti-mouse TNF-*α* PE (Table S3). Data were acquired using the NovoCyte Quanteon flow cytometer (Agilent Technologies, Waldbronn, Germany) and analysed using FlowJo software version 10.10 (FlowJo LLC, Ashland, OR, USA). The gating strategy for the analysis of the ICS data is shown in Fig. S1. Prior to determining the frequency and absolute number of cytokine-positive cells, the mock control of each animal was subtracted. Results are shown as the frequency of cytokine-producing cells in the relevant T cell compartment and the absolute numbers.

### Statistical analysis

Prism 5 (GraphPad Software Inc., Boston, MA, USA) was used for statistical analysis. Non-parametric statistical tests were used due to the presence of outliers in some data sets and the low number of data points in some groups. ELISpot and ICS data were analysed by Mann–Whitney test to compare MVA control and vaccine groups. The threshold for statistical significance was *P*<0.05.

## Results

### Generation of recombinant MVA-GP/MVA-NP expressing LASV-NP/LASV-GP genes

**Fig. 1.**
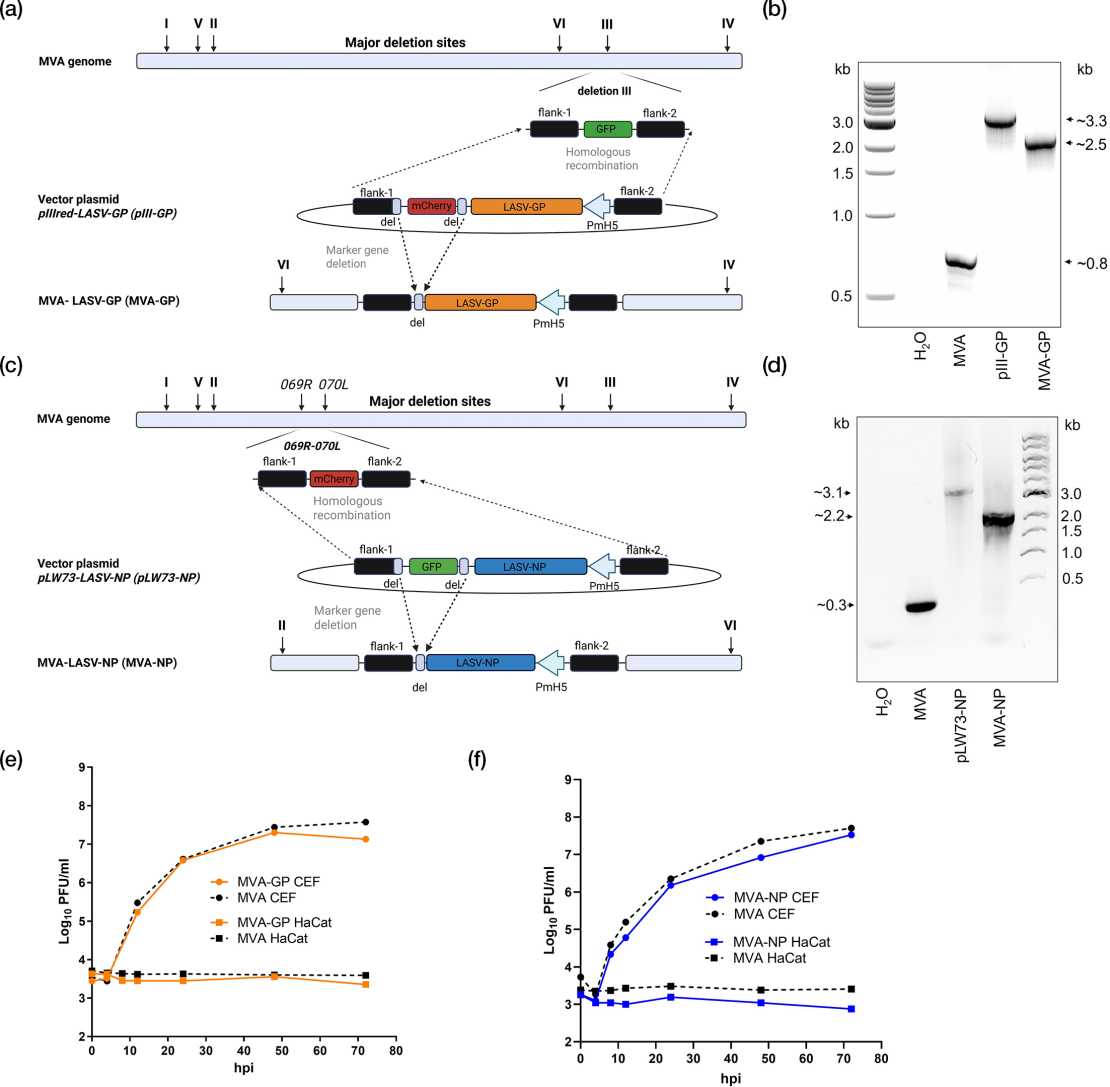
Construction and virological characterization of recombinant MVA-LASV-GP (MVA-GP) and MVA-LASV-NP (MVA-NP). (**a, c**) Scheme of the genome maps of recombinant MVA-GP and MVA-NP expressing GP and NP from LASV lineage IV and VII, respectively. LASV-GP or LASV-NP genes were inserted (**a**) into the deletion site III or (**c**) between the ORFs of the essential viral genes, *MVA069R* and *MVA070L*, as indicated. Created with BioRender.com. (**b, d**) PCR analysis of MVA-GP and MVA-NP. Viral DNA was extracted from CEF cells infected with MVA-GP, MVA-NP or non-recombinant MVA (MVA). Oligonucleotide sequences spanning the flanking regions between (**b**) the deletion site III or (**d**) *MVA069R/MVA070L* were used for PCR analysis of the GP and NP genes inserted into deletion site III or between *MVA069R/MVA070L*. Sizes of amplified DNA products are indicated with arrows on the (**b**) right or (**d**) left side. (**e, f**) Viral growth kinetics of recombinant (**e**) MVA-GP or (**f**) MVA-NP. Monolayers of CEF or HaCat cells were infected at 0.05 p.f.u. with recombinant MVA-NP, MVA-GP or non-recombinant MVA (MVA). At different time points (0, 4, 8, 12, 24, 48 and 72 hpi), cell cultures were collected and viral titres were determined by plaque assay using an anti-vaccinia virus-specific antibody.

cDNA containing the coding gene sequence of LASV-GP from the Josiah strain (lineage IV) was placed under transcriptional control of the synthetic VACV-specific early/late promoter PmH5 in the MVA vector plasmid pIII-LASV-GP (pIII-GP) and was introduced into deletion site III of MVA-GFP [67] by homologous recombination (Fig. 1a). Clonal recombinant MVA-LASV-GP (MVA-GP) was isolated in consecutive rounds of single plaque purification using the co-expression of the red fluorescent marker protein mCherry for distinction between the backbone virus MVA-GFP [67] and recombinant MVA-GP. PCR analysis of isolated viral DNA demonstrated site-specific insertion of the LASV-GP gene sequence into the MVA genome, removal of the marker protein mCherry during virus amplification (Fig. 1b) and genetic stability of the virus (Fig. S2a–c).

cDNA containing the coding gene sequence of LASV-NP from the Togo strain (lineage VII) was placed under transcriptional control of the synthetic VACV-specific early/late promoter PmH5 in the MVA vector plasmid pLW73-LASV-NP (pLW73-NP) and was introduced between the ORFs of the two MVA genes, *MVA069R* and *MVA070L*, within the MVA genome by homologous recombination (Fig. 1c) [67]. Clonal recombinant MVA-LASV-NP (MVA-NP) was isolated in consecutive rounds of single plaque purification using the co-expression of the green fluorescent marker protein GFP for distinction between the backbone virus MVA-mCherry [67] and recombinant MVA-NP. PCR analysis of isolated viral DNA confirmed site-specific insertion of the LASV-NP gene sequence into the MVA genome, removal of the marker protein GFP during virus amplification (Fig. 1d) and genetic stability of the virus (Fig. S2d–f).

The suitability of recombinant MVA-GP and MVA-NP for use under BSL-1 was confirmed by demonstrating their replicative deficiency in the human HaCat cell line (Fig. 1e, f). Additionally, the suitability of recombinant MVA-GP and MVA-NP for vaccine manufacturing at industrial scale was confirmed by demonstrating their replicative capacity in CEF cells (Fig. 1e, f). The expression of LASV-NP and LASV-GP did not impair replication of recombinant MVA-NP or MVA-GP under permissive conditions, as the results obtained from the viral growth kinetics were comparable between recombinant MVAs and non-recombinant MVA (Fig. 1e, f).

### Unimpaired expression of LASV-GP/LASV-NP in MVA-GP/MVA-NP-infected cell culture

**Fig. 2.**
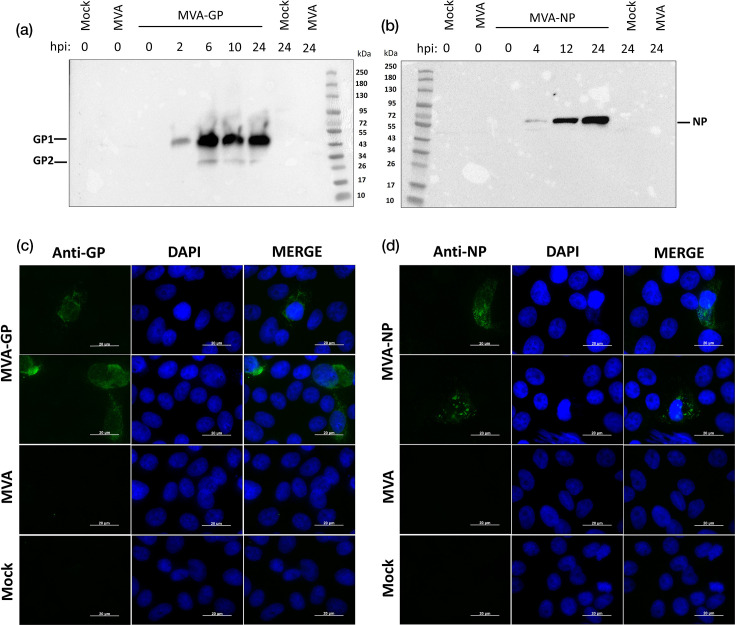
Synthesis of full-length LASV GP and NP in MVA-LASV-GP (MVA-GP) and MVA-LASV-NP (MVA-NP)-infected cells. (**a, b**) CEF cells were infected at an MOI of 10, and cell lysates were prepared after incubation for the indicated time points. Polypeptides in cell lysates were separated by SDS-PAGE and analysed with primary antibodies targeting (**a**) LASV-GP or (**b**) LASV-NP. Lysates from non-infected cells (Mock) and cells infected with non-recombinant MVA (MVA) served as controls. (**c, d**) Infected and permeabilized Vero E6 cells were incubated with polyclonal antibodies directed against the (**c**) GP (1:5,000) or (**d**) NP (1:5,000) of LASV. Polyclonal anti-rabbit antibody served for GP- or NP-specific fluorescent staining (green). Cell nuclei were counterstained with DAPI (blue). Scale bar: 20 µm.

Next, we characterized the synthesis of recombinant LASV-GP and LASV-NP expressed by MVA in infected cells by Western blotting (Fig. 2a, b) and immunofluorescence staining (Fig. 2c, d). Whole cell lysates from MVA-GP or MVA-NP-infected CEF cells were prepared for subsequent immunoblot assay. Using a polyclonal antibody directed against GP, we detected two prominent bands migrating with molecular masses of ~44 kDa and ~36 kDa [16] (Fig. 2a), representing GP1 and GP2 subunits, respectively. The expression of recombinant LASV-GP was already detectable at 2 hpi, demonstrating that the synthetic MVA-specific promoter PmH5 drove proper early transcription. Using a polyclonal antibody directed against LASV-NP, we detected a prominent band migrating with a molecular mass of ~62 kDa (Fig. 2b). The expression of recombinant LASV-NP was already detectable at 4 hpi, demonstrating that the synthetic MVA-specific promoter PmH5 drove proper early transcription. To analyse and quantify the cellular localization of LASV-GP and LASV-NP, we performed immunostaining of Vero E6 cells infected with MVA-GP or MVA-NP. Twenty-four hours post-infection, we used polyclonal antibodies against LASV-GP or LASV-NP in fixed and permeabilized cells and analysed positively stained cells with fluorescence microscopy. We observed a similar staining pattern in intracellular components of MVA-GP (Fig. 2c) and MVA-NP-infected cells (Fig. 2d).

### Recombinant MVA-GP induces LASV-GP-specific cellular immune responses in humanized HLA-A*0201/DR1 transgenic mice

**Fig. 3.**
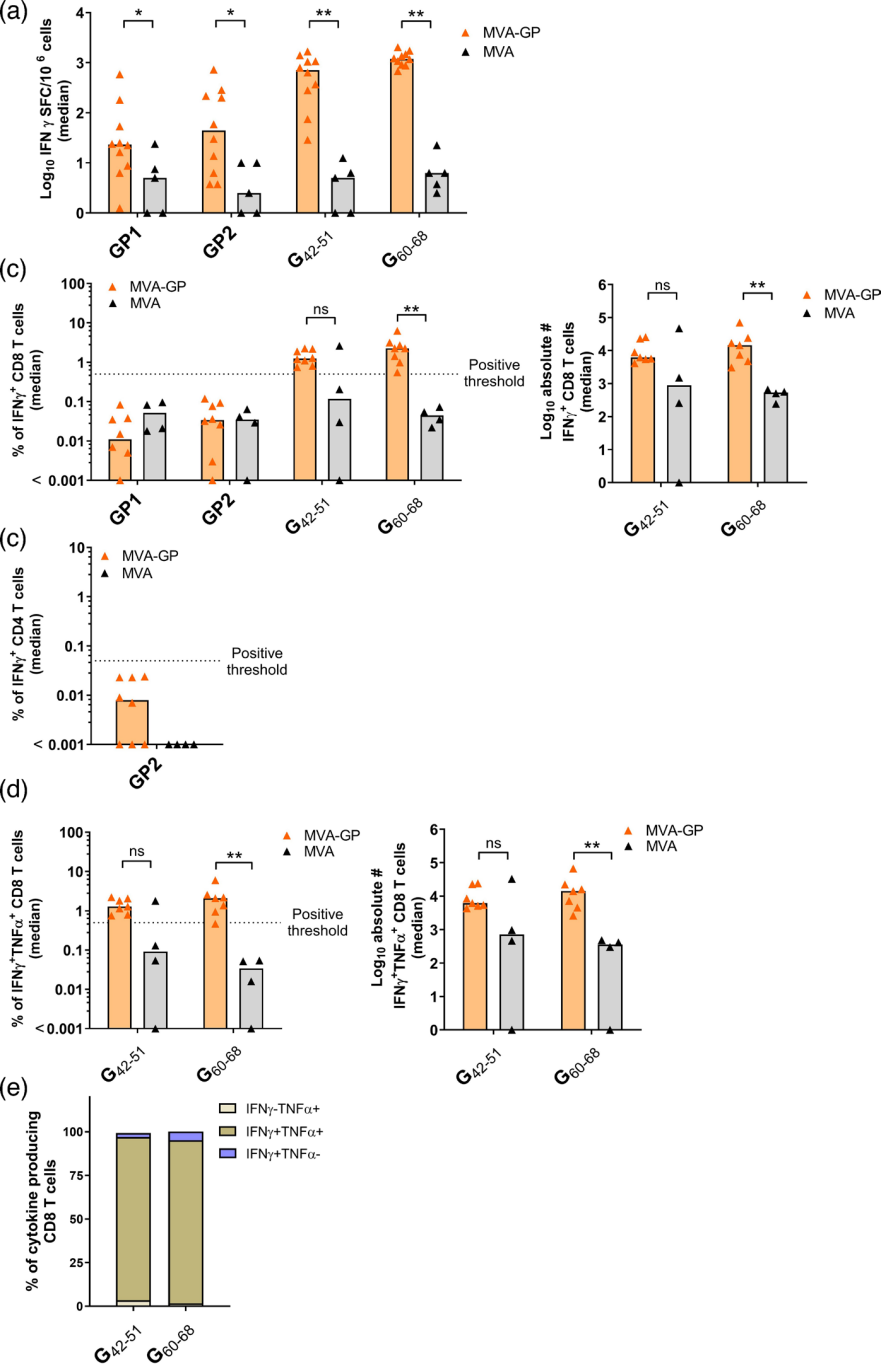
LASV-GP-specific CD8^+^ T cell responses in HLA-A*0201/DR1 transgenic mice. Groups of mice (*n*=4–10) were immunized twice with 10^7^ of MVA-LASV-GP (MVA-GP) using the IM route, and splenocytes were isolated on day 35 post-prime immunization, re-stimulated with pools of peptides or individual peptides as indicated and analysed by IFN-*γ* ELISpot assay and/or IFN-*γ*/TNF-*α* ICS plus FACS analysis. (**a**) IFN-*γ* SFC for stimulated splenocytes measured by ELISpot assay (*n*=10 for the MVA-GP vaccine group; *n*=5 for the MVA control group). (**b**) IFN-*γ* production by CD8^+^ T cells measured by ICS plus FACS analysis. Graphs show the frequency (left panel) and absolute number (right panel) of IFN-*γ*^+^ CD8+ T cells (*n*=8 for the MVA-GP vaccine group; *n*=4 for the MVA control group). (**c**) IFN-*γ*-producing CD4^+^ T cells measured by ICS plus FACS analysis. Graphs show the frequency of IFN-*γ*^+^ CD4+ T cells (*n*=8 for the MVA-GP vaccine group; *n*=4 for the MVA control group). (**d**) IFN-*γ* and TNF-*α* production by CD8^+^ T cells measured by ICS plus FACS analysis. Graphs show the frequency (left panel) and absolute number (right panel) of IFN-*γ*^+^ and TNF-*α*^+^ CD8+ T cells (*n*=7 for the MVA-GP vaccine group; *n*=4 for the MVA control group). (**e**) Cytokine profile of G_42-51_ and G_60-68_-specific CD8^+^ T cells (*n*=8 for the MVA-GP vaccine group). Graphs show the median frequency of IFN-*γ*^−^TNF-*α*^+^, IFN-*γ*^+^TNF-*α*^+^ and IFN-*γ*^+^TNF-*α*^−^ cells within the cytokine-positive CD8^+^ T cell compartment. The dashed lines indicate the threshold for positivity (0.05%). Statistical significance was determined for differences between the non-recombinant MVA and recombinant MVA-GP-vaccinated mice for each positive peptide or peptide pool by a two-tailed Mann–Whitney test. **P<0.01; *P<0.05; ns, not significant.

The activation of LASV-GP-specific cellular immune responses was characterized in humanized HLA-A*0201/DR1 transgenic mice. Mice were immunized twice over a 3-week interval with 10^7^ p.f.u. of MVA-GP using the IM administration route (Fig. S3a). We monitored the mice daily and did not observe clinical abnormalities or changes in body weight, which demonstrated good tolerability of the vaccine (Fig. S3b). Thirty-five days after initial vaccination, all mice were euthanized, and spleens were removed for further analysis. Splenocytes were isolated and stimulated with LASV-GP-specific peptides and analysed in IFN-*γ* ELISpot assay and/or ICS-FACS analysis (Fig. 3). We used a prediction software to identify potential T cell epitopes within the GP1 and GP2 subunits, and additionally, we used peptides for stimulation based on already published LASV-GP epitopes.

Prime-boost immunizations with MVA-GP generated IFN-*γ-*producing, GP-specific T cells in the spleen. Re-stimulation of isolated splenocytes with pools GP1 (G_434-442_: FVFSTSFYL, G_441-449_: YLISIFLHL) [40,78], GP2 (G_240-254_: GYLGLLSQRTRDIYI, G_431-445_: VDLFVFSTSFYLISI) [78] and the individual peptides G_42-51_ (GLVGLVTFLL) [40,78] and G_60-68_ (SLYKGVYEL) [40,65], stimulated IFN-*γ* SFC above the MVA control background [median (min–max): 23 (1–585) SFC/10^6^ cells for pool GP1; 44 (4–724) SFC/10^6^ cells for pool GP2; 713 (29–1,670) SFC/10^6^ cells for G_42-51_; 1,196 (678–2,033) SFC/10^6^ cells for G_60-68_] (Fig. 3a). Results from the IFN-*γ* ICS-FACS assay confirmed that G_60-68_ stimulated GP-specific CD8^+^ T cells (Fig. 3b), with frequencies of IFN-*γ-*producing antigen-specific CD8^+^ T cells of 2.27%. We also observed GP-specific CD8^+^ T cell responses after restimulation with G_42-51_, with frequencies of IFN-*γ-*producing antigen-specific CD8^+^ T cells of 1.24%. However, the response was statistically not significant as we observed a high background response from the MVA control group (Fig. 3b). In addition, when screening for CD4^+^ T cell responses, we did not observe IFN-*γ* positive T cells after restimulation with pool GP2, which contained the two potential CD4^+^ T cell epitope sequences GP_240-254_ (GYLGLLSQRTRDIYI) and GP_431-445_ (VDLFVFSTSFYLISI) (Fig. 3c). When we screened for co-production of TNF-*α*, we observed that the majority of G_42-51_ and G_60-68_ stimulated CD8^+^ T cells produced both IFN-*γ* and TNF-*α* [94% (G_42-51_) and 93% (G_60-68_) of the responding CD8 T cells], suggesting that the vaccine stimulated GP-specific polyfunctional CD8^+^ T cells (Fig. 3d, e).

### Recombinant MVA-NP induces LASV-NP-specific cellular immune responses in humanized HLA-A*0201/DR1 transgenic mice

**Fig. 4.**
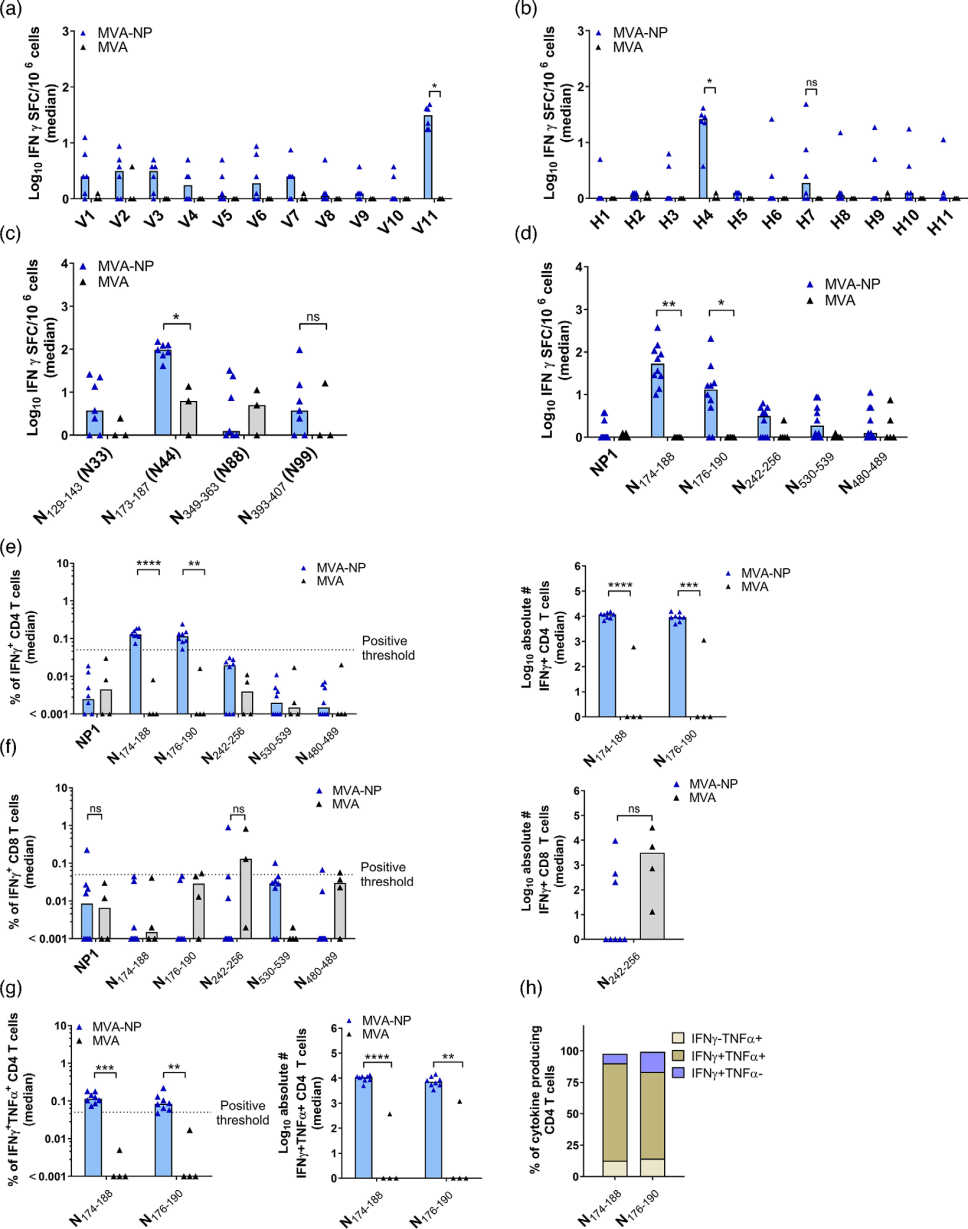
LASV-NP-specific CD4^+^ T cell responses in HLA-A*0201/DR1 transgenic mice. Groups of mice (*n*=4–10) were immunized twice with 10^7^ of MVA-LASV-NP (MVA-NP) using the intramuscular route, and splenocytes were isolated on day 35 post-prime immunization, re-stimulated with pools of peptides or individual peptides as indicated and analysed by IFN-*γ* ELISpot assay and/or IFN-*γ*/TNF-*α* ICS plus FACS analysis. (**a–d**) IFN-*γ* SFC for stimulated splenocytes measured by ELISpot assay. (**a, b**) Splenocytes were re-stimulated with pools of overlapping peptides covering the entire LASV-NP sequence (*n*=6 for the MVA-NP vaccine group; *n*=3 for the MVA control group). (**c**) Splenocytes were restimulated with individual peptides N_129-143_, N_173-187_, N_349-363_ and N_393-407_ (*n*=7 for the MVA-NP vaccine group; *n*=3 for the MVA control group). (**d**) Splenocytes were re-stimulated with a pool of NP1 or the individual peptides N_174-188_, N_176-190_, N_242-256_, N_530-539_ and N_480-489_ (*n*=10 for the MVA-NP vaccine group; *n*=5 for the MVA control group). (**e**) IFN-*γ* production by CD4^+^ T cells measured by ICS plus FACS analysis. Graphs show the frequency (left panel) and absolute number (right panel) of IFN-*γ*^+^ CD4^+^ T cells (*n*=8 for the MVA-NP vaccine group; *n*=4 for the MVA control group). (**f**) IFN-*γ*-producing CD8^+^ T cells measured by ICS plus FACS analysis. Graphs show the frequency (left panel) and absolute number (right panel) of IFN-*γ*^+^ CD8+ T cells (*n*=8 for the MVA-NP vaccine group; *n*=4 for the MVA control group). (**g**) IFN-*γ* and TNF-*α* production by CD4^+^ T cells measured by ICS plus FACS analysis. Graphs show the frequency (left panel) and absolute number (right panel) of IFN-*γ*^+^ and TNF-*α*^+^ CD4+ T cells (*n*=8 for the MVA-NP vaccine group; *n*=4 for the MVA control group). (**h**) Cytokine profile of N_174-188_ and N_176-180_-specific CD4^+^ T cells. Graphs show the frequency of IFN-*γ*^−^TNF-*α*^+^, IFN-*γ*^+^TNF-*α*^+^ and IFN-*γ*^+^TNF-*α*^−^ cells within the cytokine-positive CD8^+^ T cell compartment (*n*=8 for the MVA-NP vaccine group). The dashed lines indicate the threshold for positivity (0.05%). Statistical significance was determined for differences between the non-recombinant MVA and recombinant MVA-NP vaccinated mice for each positive peptide or peptide pool by a two-tailed Mann–Whitney test. **P<0.01; *P<0.05; ns, not significant.

The activation of LASV-NP-specific cellular immune responses was characterized in humanized HLA-A*0201/DR1 transgenic mice. Mice were immunized twice over a 3-week interval with 10^7^ p.f.u. of MVA-NP using the IM administration route (Fig. S3a). We monitored the mice daily and did not observe clinical abnormalities or changes in body weight, which demonstrated good tolerability of the vaccine (Fig. S3b). Thirty-five days after initial vaccination, all mice were euthanized, and spleens were removed for further analysis. Splenocytes were isolated and stimulated with LASV-NP-specific peptides and analysed in IFN-*γ* ELISpot assay and/or ICS-FACS analysis (Fig. 4). To make the best possible use of all tentative LASV-NP-specific T cell epitopes, we used pools of overlapping peptides covering full-length LASV-NP, pooled in a two-dimensional, pooled-peptide matrix system. We also used peptides for stimulation based on a prediction software and already published LASV-NP epitopes.

Prime-boost immunizations with MVA-NP stimulated LASV-NP-specific T cells in the spleen. Isolated splenocytes were stimulated with pools of 15-mer peptides (N1–N140), overlapping by 11 mer, that spanned the entire LASV-NP sequence (Table S2). After restimulation with pools V1–V10, only weak responses were detected, with median counts between 1 and 4 SFU/10^6^ cells. In contrast, restimulation with pool V11 induced a robust antigen-specific T cell response [median (min–max): 31 (18–49) SFU/10^6^ cells] (Fig. 4a). After restimulation with pools H1-H3, H5, H6 and H8-H11, only weak responses were detected, with median counts between 1 and 2 SFU/10^6^ cells. In contrast, restimulation with pools H4 and H7 induced a robust antigen-specific T cell response [median (min–max): 26 (4–41) SFU/10^6^ cells for H4 and 2 (0–48) SFU/10^6^ cells for pool H7] (Fig. 4b). For subsequent analysis, we focused on the three pools V11, H4 and H7, which induced the highest antigen-specific T cell responses.

When comparing the peptide sequences, we identified four peptides (N33, N44, N88 and N99) included twice within the three pools, indicating that these sequences might be part of potential NP-specific T cell epitopes. Interestingly, computational analysis identified the amino acid region around peptide N44 (NNQFGTMPSLTLACL) [59] as a putative LASV-NP-specific T cell epitope, which perfectly matched our observations when testing the individual peptides N33, N44, N88 and N99 in the ELISpot assay [median (min–max): 4 (0–26) SFU/10^6^ cells for N33; 96 (41–151) SFU/10^6^ cells for N44; 1 (0–33) SFU/10^6^ cells for N88; and 4 (0–98) SFU/10^6^ cells for N99] (Fig. 4c). After re-stimulation of isolated splenocytes with pool NP1 (N_38-47_: LLHGLDFSEV; N_364-373_: GLTYSQLMTL) [40,78] or the individual peptides N_530-539_ (ALLDCIMFDA) [40,78], N_480-489_ (KLIDVSLNKI), N_174-188_ (NQFGTMPSLTLACLT) [61], N_242-256_ (SGYNFSLGAAIKTGA) [78] and N_176-190_ (FGTMPSLTLACLTKQ) [61], we observed IFN-*γ* SFC above the MVA control background for peptides N_174-188_ (NQFGTMPSLTLACLT) [61] and N_176-190_ (FGTMPSLTLACLTKQ) [61] [median (min–max): 54 (10–377) SFU/10^6^ cells for N_174-188_ and 13 (0–209) SFU/10^6^ cells for N_176-190_] (Fig. 4d). The IFN-*γ* ICS assay confirmed that N_174-188_ and N_176-190_ stimulated LASV-NP-specific CD4^+^ T cells (Fig. 4e), rather than CD8^+^ T cells (Fig. 4f). The frequency of IFN-*γ-*producing antigen-specific CD4^+^ T cells was 0.13% for N_174-188_ and 0.12% for N_176-190_. When we screened for co-production of TNF-*α*, we observed that the majority of N_174-188_ and N_176-190_ stimulated CD4^+^ T cells produced both IFN-*γ* and TNF-*α* (78% for N_174-188_ and 69% for N_176-190_), suggesting that the vaccine stimulated NP-specific polyfunctional CD4^+^ T cells (Fig. 4g, h).

### Recombinant MVA-GP and MVA-NP vaccine candidates induce VACV-specific cellular immune responses in humanized HLA-A*0201/DR1 transgenic mice

**Fig. 5.**
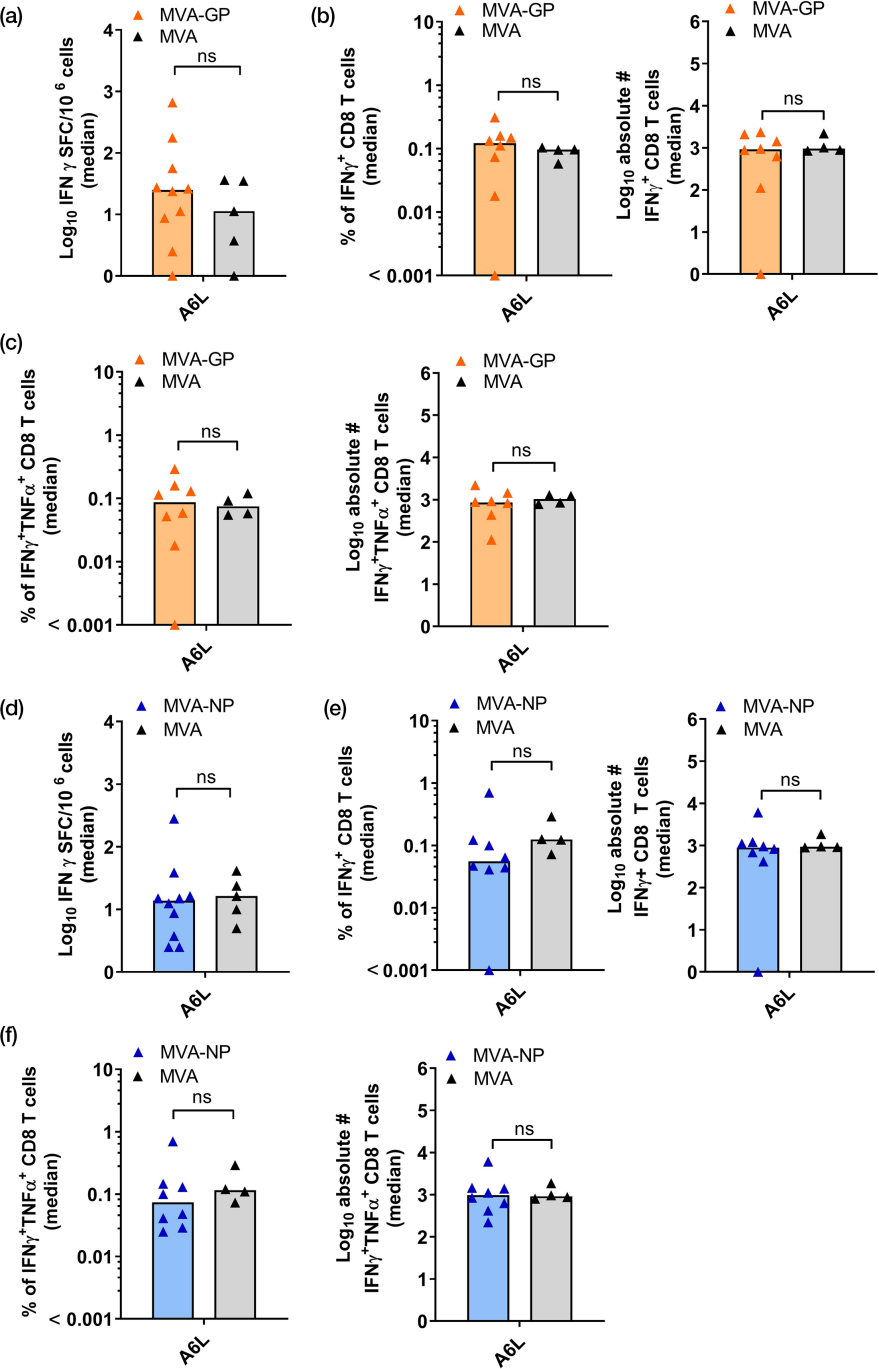
MVA-specific CD8^+^ T cell responses in HLA-A*0201/DR1 transgenic mice following vaccination with MVA-LASV-GP (MVA-GP) and MVA-LASV-NP (MVA-NP). Groups of mice (*n*=4–10) were immunized twice with recombinant (**a–c**) MVA-GP, (d**–f**) MVA-NP or (**a–f**) non-recombinant MVA (MVA) as control. Splenocytes were collected on day 35 post-prime immunization, re-stimulated with peptide A6L_6-14_ and analysed by IFN-*γ* ELISpot assay and/or IFN-*γ*/TNF-*α* ICS plus FACS analysis. (**a, d**) IFN-*γ* SFC for stimulated splenocytes measured by ELISpot assay. (**b, e**) IFN-*γ* production by CD8^+^ T cells were measured by ICS plus FACS analysis. Graphs show the frequency (left panel) and absolute number (right panel) of IFN-γ^+^ CD8+ T cells. (**c, f**) IFN-*γ*^+^/TNF-*α*^+^ splenocytes after re-stimulation measured by ICS plus FACS analysis. Graphs show the frequency (left panel) and absolute number (right panel) of IFN-*γ*^+^ CD8+ T cells. Statistical significance was determined for differences between the non-recombinant MVA and recombinant MVA-NP vaccinated by a two-tailed Mann–Whitney test. ns, not significant.

Next, we explored the VACV-specific T cell responses induced by MVA-GP and MVA-NP (Fig. 5). Isolated splenocytes were re-stimulated with the VACV-specific peptide A6L_6-14_ (VLYDEFVTI) [76], and the activation of T cells was measured by IFN-*γ* ELISpot assay (Fig. 5a, d) and IFN-*γ*/TNF-*α* ICS assay (Fig. 5b, c, e and f). The ELISpot assay confirmed the induction of IFN-*γ* positive T cells for the MVA-GP vaccine group [median (min–max): 25 (0–659) SFC/10^6^ cells] (Fig. 5a) and for the MVA-NP vaccine group [median (min–max): 14 (3–280) SFC/10^6^ cells] (Fig. 5d). The IFN-*γ* ICS assay confirmed that re-stimulation of isolated splenocytes with A6L_6-14_ stimulated the induction of CD8^+^ T cells. The frequencies of IFN-*γ-*producing antigen-specific CD8^+^ T cells were 0.12% (MVA-GP, Fig. 5b) and 0.06% (MVA-NP, Fig. 5f). The frequencies of both IFN-*γ-* and TNF-*α-*producing antigen-specific CD8^+^ T cells were 0.08% (MVA-GP, Fig. 5c) and 0.08% (MVA-NP, Fig. 5f). Overall, we did not detect differences in the induction of vector-specific immune responses between the vaccine group and the MVA control group.

## Discussion

Despite ongoing research on developing medical countermeasures, neither effective vaccines nor specific antiviral treatment is available against LASV. Several groups worked on the generation of effective vaccines, predominantly focusing on NP and/or GP from LASV strains belonging to lineages III and IV [36,42,44, 45, 47]. However, due to the inter-lineage diversity (Tables S4 and S5) between some LASV strains, vaccines might be less effective against other circulating or newly emerging LASV strains. Furthermore, this inter-lineage diversity is associated with differences in pathogenicity as seen in infection studies using non-human primates. Cynomolgus monkeys, which fully recapitulate LF in humans, succumbed to infection with a LASV strain from lineage VII, accompanied by viral replication, increased inflammatory response and absence of efficient immune responses [79], which resembled the pathogenesis upon infection with the prototype Josiah strain (lineage IV) [80]. In contrast, infection with LASV strains from lineages II or V was associated with lower mortality rates, efficient T cell responses [79] and atypical clinical manifestations such as moderate to severe pulmonary symptoms [81].

Interestingly, a cellular-mediated immune response is thought to be a key factor contributing to the protective immunity against LASV. The activation of an early cellular immune response (both CD4^+^ and CD8^+^ T cells) was associated with LASV clearance and recovery in both human and non-human primates, which supports the important role of cellular immune responses in viral clearance [44,45, 60, 82]. In addition, low levels of T cells and a delay in activation of cellular immune responses have been found in patients with a fatal LF disease outcome [61,83]. Vaccination studies with recombinant VACV or MVA expressing internal proteins such as NP, which are thought to induce cellular immunity rather than humoral immunity, protected against lethal LASV infection in guinea pigs [39,41]. With regard to innovative vaccination strategies, targeting dominant, pronounced activation of vaccine-induced T cells might result in a rapid (within a few days), broader-reactive and long-lasting protection against LASV infections. This has been confirmed in previous studies [36,84, 85]. Geisbert *et al.* [84] generated a VSV-LASV vaccine expressing full-length glycoprotein from the Lassa Josiah strain (lineage IV), which protected cynomolgus macaques against infection with the LASV Josiah strain when administered as a single-dose vaccine 28 days before challenge. This protection was associated with the induction of LASV-GP-specific CD8^+^ T cells and LASV-GP-specific humoral immune responses. In addition, Cross *et al.* [36] have shown that the VSV-LASV vaccine protected cynomolgus monkeys against a lethal infection with a LASV strain from lineage II, when vaccinated three or 7 days before challenge. Furthermore, the same group [85] developed a quadrivalent vaccine consisting of VSV vectors expressing glycoproteins of EBOV, Sudan ebolavirus (SUDV), Marburg virus (MARV) and LASV Josiah strain (lineage IV). Prime-boost vaccinations 8 weeks apart induced a strong T cell response against all four glycoproteins and protected cynomolgus macaques against lethal challenge with EBOV, SUDV, MARV and a LASV strain from lineage II.

To investigate the capability of MVA-based vaccines to induce LASV-specific T cells, we generated two vaccines targeting GP from the prototype Josiah strain (lineage IV) and NP from the recently discovered Togo strain (lineage VII) and explored their immunogenic properties in HLA-A*0201/DR1 transgenic mice. HLA-A*0201/DR1 transgenic mice express human leucocyte antigen (HLA) class I/II molecules (HLA-A*02:01/HLA-DRB1*01:01); thus, immunization of this mouse strain allows for the identification of HLA-restricted CD4^+^ and CD8^+^ epitopes [86]. Our study confirmed that the recombinant MVA-GP and MVA-NP vaccines significantly stimulated IFN-*γ* and TNF-*α* production in vaccinated mice after two immunizations. The up-regulation of a cellular-mediated immune response was in agreement with published data from other MVA-based vaccines targeting LASV [41,42]. Kennedy *et al.* [41] generated an MVA-based vaccine targeting NP from a LASV strain belonging to lineage III and confirmed the induction of cellular immunity in mice and guinea pigs. Furthermore, NP was sufficient to protect guinea pigs in a LASV challenge model. Salvato *et al.* [42] generated an MVA-based vaccine expressing both GP and Z-antigens from the LASV Josiah strain, which induced the formation of virus-like particles in cell culture, and confirmed protective efficacy in CBA/J mice against an attenuated Mopeia–Lassa reassortant. The authors detected both CD8^+^ and CD4^+^ T cell responses after re-stimulation of splenocytes with G_60-68_ (SLYKGVYEL), G_441-449_ (YLISIFLHL) and G_42-51_ (GLVGLVTFLL). In agreement with these results, we detected high levels of LASV-GP-specific polyfunctional CD8^+^ T cells when we used the same peptides (G_60-68_, G_441-449_ and G_42-51_), in addition to peptides G_434-442_ (pool GP1; FVFSTSFYL), G_240-254_ (pool GP2; GYLGLLSQRTRDIYI) and G_431-445_ (pool GP2; VDLFVFSTSFYLISI) [78] to stimulate splenocytes from MVA-GP vaccinated mice (Fig. 3). The location of immunogenic epitopes induced by vaccination with MVA-GP was found within the SSP/GP1 subunit (G_60-68_ and G_42-51_) and the GP2 subunit (G_441-449_, G_434-442_, G_431-445_ and G_240-254_) (Figs S4 and 3), suggesting that the full-length GP should be used for vaccine design to induce a broader T cell response. More precisely, both of these epitopes are located within the receptor binding domain (RBD) of GP, which in principle is a highly conserved region to preserve the receptor binding function of RBD to initiate the essential entry process. In doing so, we hypothesize that these epitopes located in functionally critical regions like the RBD are efficiently processed and bind MHC molecules with high affinity and are immunodominant. This might explain the prominent activation of T cell responses throughout our analysis compared to the GP1 and GP2 overlapping peptide pools. In doing so, T cells specific for such immunodominant and conserved epitopes may have a broader cross-reactivity and result in better control of viral infection. This makes such GP-specific T cell responses potentially protective and durable [64,87, 88]. In our studies, the GP-specific T cell responses we detected have been mainly identified as CD8^+^ T cells. The lack of sufficient levels of CD4^+^ T cells after stimulation with the GP1 and GP2 overlapping peptides might be explained by the narrow peptide pools, which consist of 15-mer overlapping by 11 peptides. Such a peptide pool might miss optimal epitope processing or presentation windows for CD4^+^ T cells, since CD4^+^ T cells respond best to longer or specific-length peptides (e.g. 18–25-mers). For this, future studies using a more targeted or refined peptide mapping approach could reveal stronger or more consistent CD4^+^ T cell responses [89]. Our findings with MVA-GP and MVA-NP in the HLA-A*0201/DR1 transgenic mouse model – demonstrating strong cellular immune activation – are consistent with prior reports from other platforms, including VSV-based and MV-based LASV vaccine candidates, which also elicit robust T cell responses [36,45, 90].

Since the use of HLA-A*0201/DR1 transgenic mice allows for the identification of HLA-restricted CD4^+^ and CD8^+^ T cell epitopes from humans, we compared the T cell epitopes identified in our study with published data. The identified region within NP, located at position N_173-190_, is in agreement with published data by ter Meulen *et al.*, who showed that seropositive individuals from endemic regions have a memory CD4^+^ T cell response specific for this LASV-NP location. In this study, PBMCs from donors were stimulated with pools of peptides, also including peptides covering the location of N_169-188_ and N_176-195_ [61] within NP, and proliferation was observed. In our study, we mainly detected LASV-NP-specific CD4^+^ T cells, but not CD8^+^ T cells, upon vaccination of HLA-A*0201/DR1 transgenic mice. Notably, across vaccine platforms including MVA-, VSV- and MV-based candidates, CD4^+^ T cell responses have generally been stronger in magnitude compared to CD8^+^ responses, indicating a prominent role of CD4^+^ T cell activation in LASV vaccine-induced immunity [41,42, 44, 84].

The lack of detectable CD8^+^ T cell responses to NP epitopes in our study, despite broad HLA class I coverage, may reflect factors such as immunodominance, inefficient antigen processing or suboptimal peptide presentation *in vitro*. The use of overlapping 15-mer peptides, while comprehensive, may not adequately stimulate CD8^+^ T cells compared to minimal optimal epitopes due to MHC I binding limitations. Although we did not directly assess cross-reactive CD8^+^ responses, our peptide selection was informed by epitope mapping in LASV survivors [64] and conservation across LASV lineages, supporting the relevance of the targets for broad vaccine coverage. Our data are in agreement with data from Sullivan *et al.* who confirmed that LF survivors from Sierra Leone and Nigeria showed a stronger LASV-NP-specific CD4^+^ than CD8^+^ T cell response [64]. Interestingly, sequence alignments with all major and proposed lineages of LASV revealed that this particular location is conserved across all lineages (Fig. S5). Sullivan *et al.* [64] also showed that LF survivors can develop polyfunctional CD4^+^ and CD8^+^ T cell responses that are cross-reactive between LASV lineages. LASV-specific T cells that were generated upon primary infection with LASV lineage II or III reacted with antigens from lineage IV and responded to both NP and GP. This was in agreement with data published by Sakabe *et al.* [65], who isolated PBMCs from LF survivors from Nigeria and Sierra Leone and re-stimulated them with recombinant VSVs encoding parts of GP and NP, confirming LASV-GP- and LASV-NP-specific CD8^+^ T cell responses in 6/6 and 4/6 individuals, respectively. When looking more into detail, 2/6 individuals responded to antigens G_1-58_ and G_34-94_, which was in agreement with our positive ELISpot and ICS-FACS results when using peptides G_42-51_ and G_60-68_. Furthermore, 6/6 individuals responded either to antigens G_194-259_ or G_240-299_, which was also in agreement with our results (Fig. 3). However, the role of the identified LASV-specific T cells in both LF survivors and immunized mice has to be evaluated in clinical trials under controlled settings.

Interestingly, the role of humoral immunity for protection against LF is still poorly understood. Some groups have shown that an induction of humoral immune responses was not sufficient to protect non-human primates against a lethal LASV infection [91,92], whereas others demonstrated that the induction of neutralizing and/or non-neutralizing antibodies is important for viral clearance [93,94]. Furthermore, it has been shown that the use of convalescent plasma and monoclonal antibodies derived from LF survivors protected cynomolgus monkeys against a lethal LASV challenge [95,97]. During acute LASV infection, no or only small amounts of binding IgG or neutralizing antibodies are detectable and appear only several weeks or months after recovery [82]. These findings are in agreement with data published by Salvato *et al.* [42], who detected LASV-GP binding antibodies in serum of CBA/J mice only after challenge infection with LASV, but not after immunization with an MVA vaccine expressing GP and Z. In contrast, Kennedy *et al.* [41] detected LASV-NP-specific binding antibodies after prime and prime-boost immunization schedules in BALB/c mice, confirming the capability of MVA-LASV-based vaccines to induce also humoral immune response. Since our major interest was to evaluate the capability of MVA-GP and MVA-NP vaccine candidates to activate LASV-epitope-specific T cells that mimic immunodominant T cell specificity also found in humans, we used in our study HLA-A*0201/DR1 transgenic mice. They are highly suitable to identify human antigen-specific T cell epitopes due to the lack of murine MHC class I/II molecules (H-2 knockout) and the expression of human MHC molecules HLA-A2.1 (class I) and HLA-DR1 (class II) [86]. Thus, these mice allow the identification of T cell responses that are restricted by human MHC molecules, particularly HLA-A2.1 (CD8^+^ T cells) and HLA-DR1 (CD4^+^ T cells), as it has been confirmed for the immunodominant HLA-A2.1-restricted VACV epitope A6L_6-14_ (VLYDEFVTI) that is recognized by cytotoxic CD8^+^ T cells [76,77]. However, since B cell development and antibody responses are mainly driven by murine factors which are incompletely expressed due to the H-2 knockout, it is hypothesized that this model is primarily suited to analyse T cell responses. In doing so, we did not analyse the activation of LASV-GP-/LASV-NP-specific antibodies in the HLA-A*0201/DR1 transgenic mouse model. Therefore, to evaluate the capability and kinetics of our recombinant MVA-GP and MVA-NP vaccines to induce humoral immunity, future studies in different animal models and non-human primates are warranted. Additional experiments will also include efficacy testing in a suitable challenge model (e.g. guinea pig or IFNAR^-/-^ mice), serum transfer and T cell depletion experiments to further characterize the role of cellular and humoral immune responses in the immunity against LASV.

Regarding HLA-A2.1 and HLA-DR1, since these alleles are predominantly found across the Caucasian population, additional studies should consist of testing for other HLA alleles that are more frequently found in the West African population. Moreover, since our data from GP indicate a polyfunctional T cell response specific for a region of the RBD that is highly conserved, we hypothesize that the MVA-GP vaccine might induce broadly protective immune responses, which might be an advantage for the current outbreak situation in Africa. Interestingly, MVA-NP also resulted in the activation of a polyfunctional and broadly reactive T cell response as seen by the activation of CD4^+^ and CD8^+^ T cells, which also implies a broadly protective immunity. Thus, it will now be of interest to directly evaluate the outcome of MVA-GP- and MVA-NP-induced protection. A special approach would then also include combining both antigens to evaluate a possible benefit for protection, especially against different strains of LASV.

MVA as a viral vector platform offers a variety of advantages, including its replication deficiency in mammalian cells, the capability to insert multiple and long DNA sequences and the genetic stability due to its nature as a DNA virus. Furthermore, MVA-based vaccines can be administered without adjuvants, which is an advantage compared to other vaccine platforms (e.g. protein subunit vaccines). The high safety profile of MVA and promising data on immunogenicity and efficacy obtained from various preclinical and clinical trials indicate that MVA might be a suitable platform to generate vaccines against LF, especially for the vulnerable population, such as pregnant women or immunocompromised individuals. Our newly generated MVA-NP and MVA-GP vaccines were proven safe and were well-tolerated upon prime-boost vaccination in rodents with no signs of clinical abnormalities or weight loss.

In addition to local outbreaks of haemorrhagic fever viruses, such as EBOV, LASV or MARV, Africa is facing a severe mpox epidemic caused by the monkeypox virus (MPXV). MVA is licensed as a vaccine against human mpox and has been confirmed to robustly protect against MPXV infections [98]. In addition, clinical trials are carried out in Africa to determine the suitability of MVA-BN in pregnant women and infants [99]. Since the target antigens LASV-NP and LASV-GP were inserted into an MVA backbone, our two candidate vaccines would cover two pathogenic viruses, LASV and MPXV, which circulate and are endemic in the same areas. In our study, we tested for the induction of anti-VACV-specific cellular immune responses after prime-boost immunizations with MVA-NP or MVA-GP and confirmed the induction of a robust VACV-specific CD8^+^ T cell response, which further supports the suitability of MVA-GP and MVA-NP candidate vaccines to serve as bivalent vaccines due to the well-established cross-reactivity within the genus *Orthopoxvirus* [100]. However, the usefulness of MVA-GP and/or MVA-NP as potential dual vaccines against Mpox and LASV has to be characterized in future studies and was beyond the scope of this study.

A notable limitation in our study is the focus on the T cell-mediated immunity to LASV-antigens NP and GP. While this first proof-of-concept study has provided first insights into the cellular immune mechanisms induced by our MVA-LASV candidate vaccines, this represents only one arm of the adaptive immune response. Humoral immunity, especially the role of neutralizing and non-neutralizing antibodies, remains underexplored in many experimental vaccine models. Given that Lassa virus glycoproteins are the primary targets of antibody responses, future studies must expand to include detailed antibody profiling – including binding specificity, neutralization capacity and Fc-mediated functions – as critical correlates of protection. A comprehensive understanding of both cellular and humoral responses will be essential to define robust and durable immunity, guiding the development of next-generation Lassa virus vaccines.

Taken together, prime-boost immunizations with recombinant MVA-GP- or MVA-NP-induced substantial levels of LASV-GP and LASV-NP epitope-specific T cell responses. The confirmed T cell epitopes were in agreement [61,78] with published human-specific LASV T cell epitopes, highlighting the suitability of using this humanized mouse model for the preclinical evaluation of candidate vaccines. The next step will be to assess the efficacy of the two vaccines in a lethal challenge model, using rodents or guinea pigs.

## Supplementary material

10.1099/jgv.0.002142 Uncited Supplementary Material 1

## Abbreviations

CEF: chicken embryonic fibroblasts
EBOV: Ebola virus
ELISpot: enzyme-linked immunospot
GP: glycoprotein
GPC: glycoprotein precursor
hpi: h post-infection
ICS: intracellular cytokine staining
IEDB: Immune Epitope Database
IF: immunofluorescence
IM: intramuscular
LASV: Lassa virus
LF: Lassa fever
MARV: Marburg virus
MEM: Minimum Essential Medium Eagle
MOI: multiplicity of infection
MPXV: monkeypox virus
MV: measles virus
MVA: modified vaccinia virus Ankara
MVA-LassNP: MVA-based LASV vaccine expressing NP
NP: nucleoprotein
PMA: phorbol-12-myristate-13-acetate
RBD: receptor binding domain
SFC: spot-forming cells
SPF: specific pathogen free
SSP: stable signal peptide
SUDV: Sudan ebolavirus
VACV: vaccinia virus
VLP: virus-like particles
VSV: vesicular stomatitis virus
