# Supplementary material for: Robust polyfunctional CD8+ and CD4+ T cell responses in HLA-A*0201/DR1 transgenic mice following vaccination with modified vaccinia virus Ankara-based vaccines delivering Lassa virus glycoprotein or nucleoprotein
Source: J Gen Virol. 2025 Sep 1;106(9):002142. doi: 10.1099/jgv.0.002142 (PMC12451764; doi:10.1099/jgv.0.002142)
Supplement: Uncited Supplementary Material 1 [file jgv-106-02142-s001.pdf]

Supplementary Data and Supplementary Tables

Tscherne *et al*. 2025

Robust polyfunctional CD8$^+$ and CD4$^+$ T Cell responses in HLA-A*0201/DR1 transgenic mice following vaccination with Modified Vaccinia virus Ankara based vaccines delivering Lassa virus glycoprotein or nucleoprotein

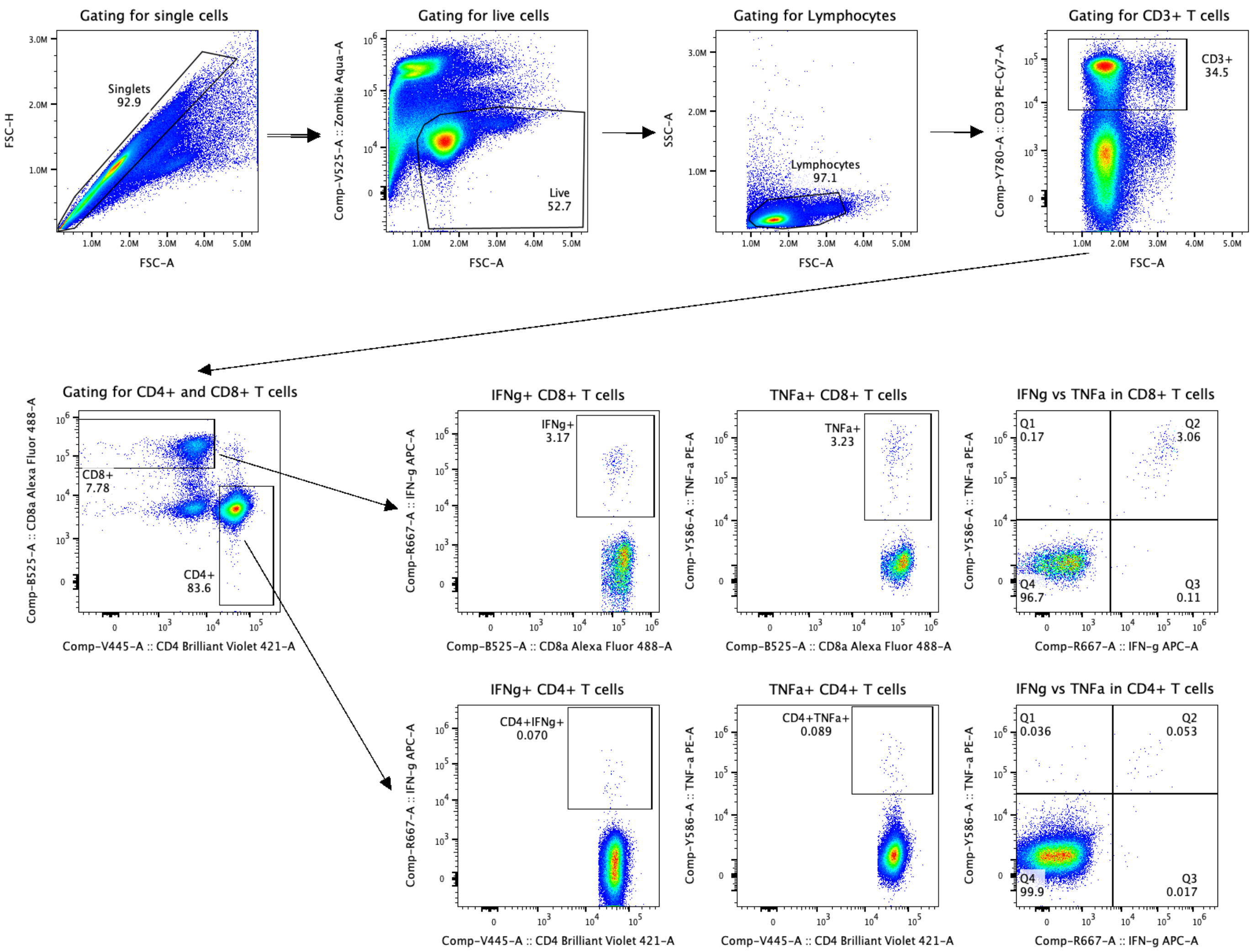

Supplementary Figure S1: Gating strategy for flow cytometric analysis. After the exclusion of doublets and dead cells, samples were gated for lymphocytes followed by CD3+ and then CD4+ and CD8+ T cells. CD4+ and CD8+ T cells were then gated for the total IFN-γ$^+$ and total TNF-α$^+$ cell population. In addition, the intracellular expression of IFN-γ and TNF-α was compared directly within each T cell compartment.

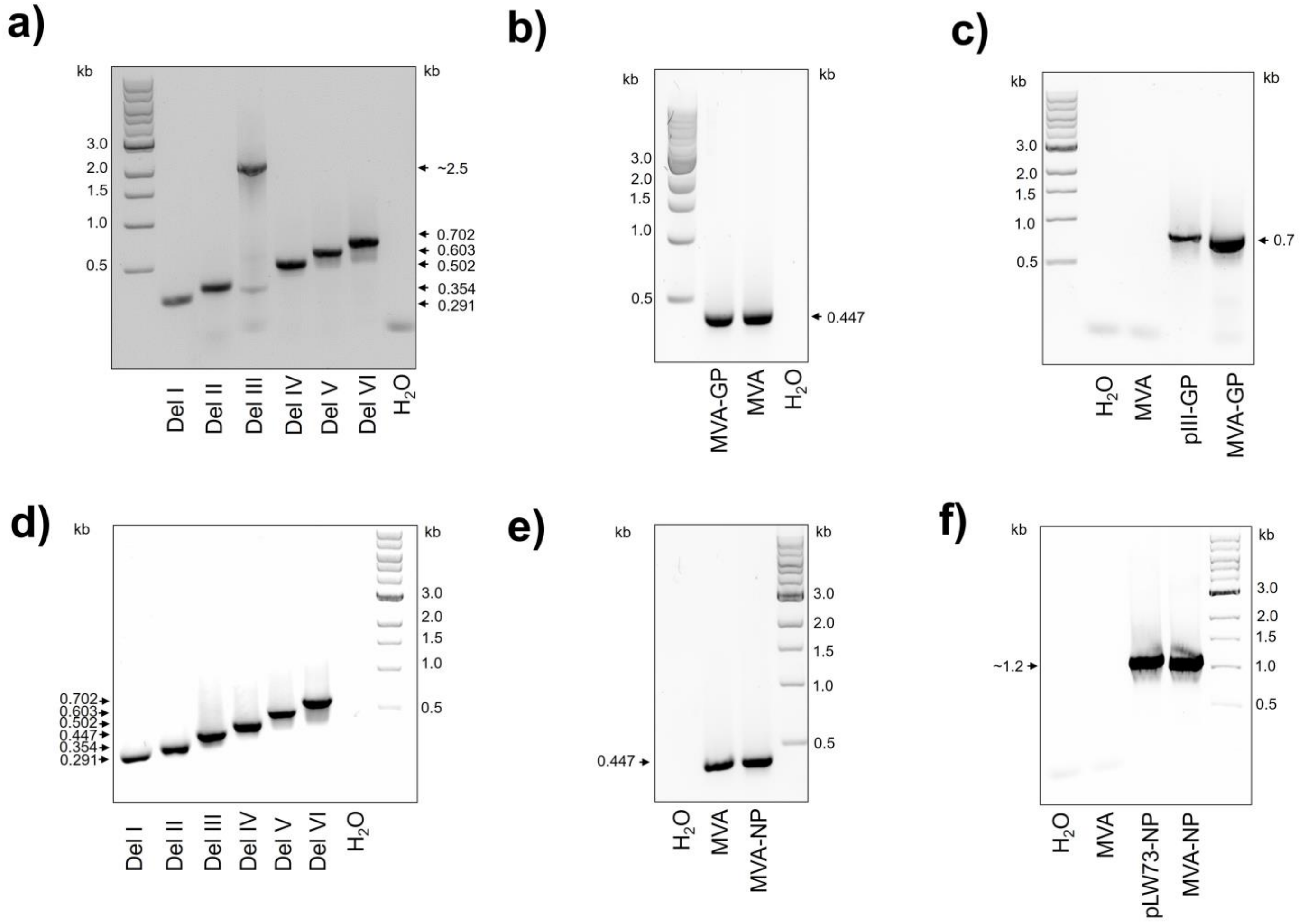

Supplementary Figure S2: PCR analysis of viral DNA to monitor genetic stability and integrity of MVA-LASV-GP (MVA-GP) **(a-c)** and MVA-LASV-NP (MVA-NP) **(d-f)**. **(a, d)** PCR with specific oligonucleotide sequences targeting the six major deletions sites (Del I-VI) of **(a)** MVA-GP and **(c)** MVA-NP confirmed genetic stability of the six loci and the correct insertion of the LASV-GP coding sequence into **(a)** deletion site III. **(b, e)** Amplification of a specific 0.447 kb DNA fragment from the MVA *C7L* gene sequence confirmed integrity of the *C7L* gene locus in the MVA-GP and MVA-NP genome. **(c)** Amplification of a specific 0.7 kb DNA fragment from the inserted LASV-GP gene sequence demonstrated genetic integrity. **(f)** Amplification of a specific 1.2 kb DNA fragment from the inserted LASV-NP gene sequence demonstrated genetic integrity.

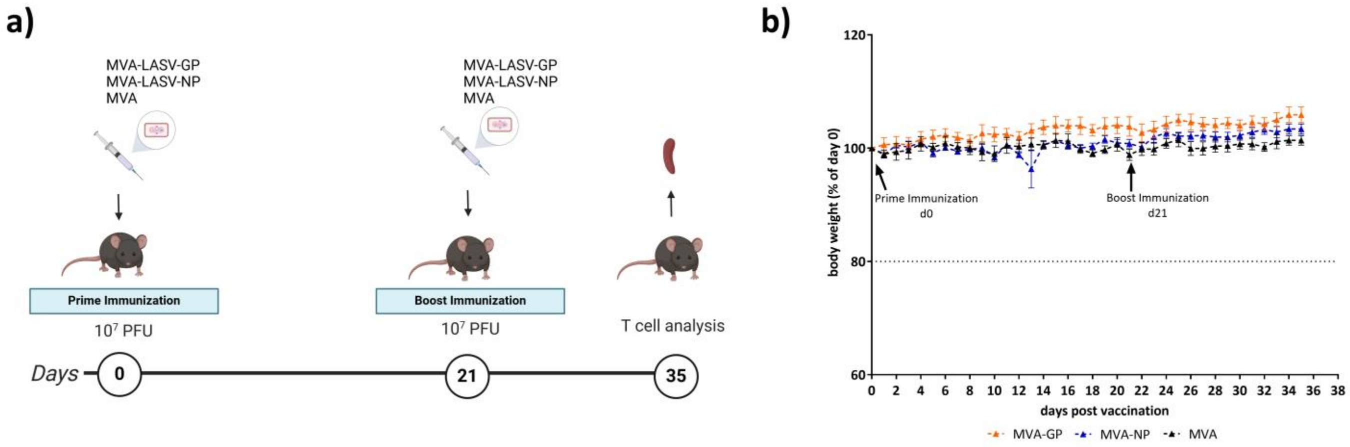

Supplementary Figure S3: (a, b) Immunization schedule and weight curves. **(a)** HLA-A*0201/DR1 transgenic mice received MVA-NP, MVA-GP or non-recombinant MVA in a prime-boost regimen using a 21-day interval. For each immunization, mice received a dose of 10^7 PFU by using the intramuscular route. Splenocytes were collected on day 35 post prime immunization. Created with BioRender.com **(b)** Body weight progression after immunization of HLA-A*0201/DR1 transgenic mice with recombinant MVA-NP or MVA-GP. The dashed line represents the maximum acceptable body weight loss (20%). Mice with a body weight loss of >20% had to be euthanized according to the predefined supervision protocol.

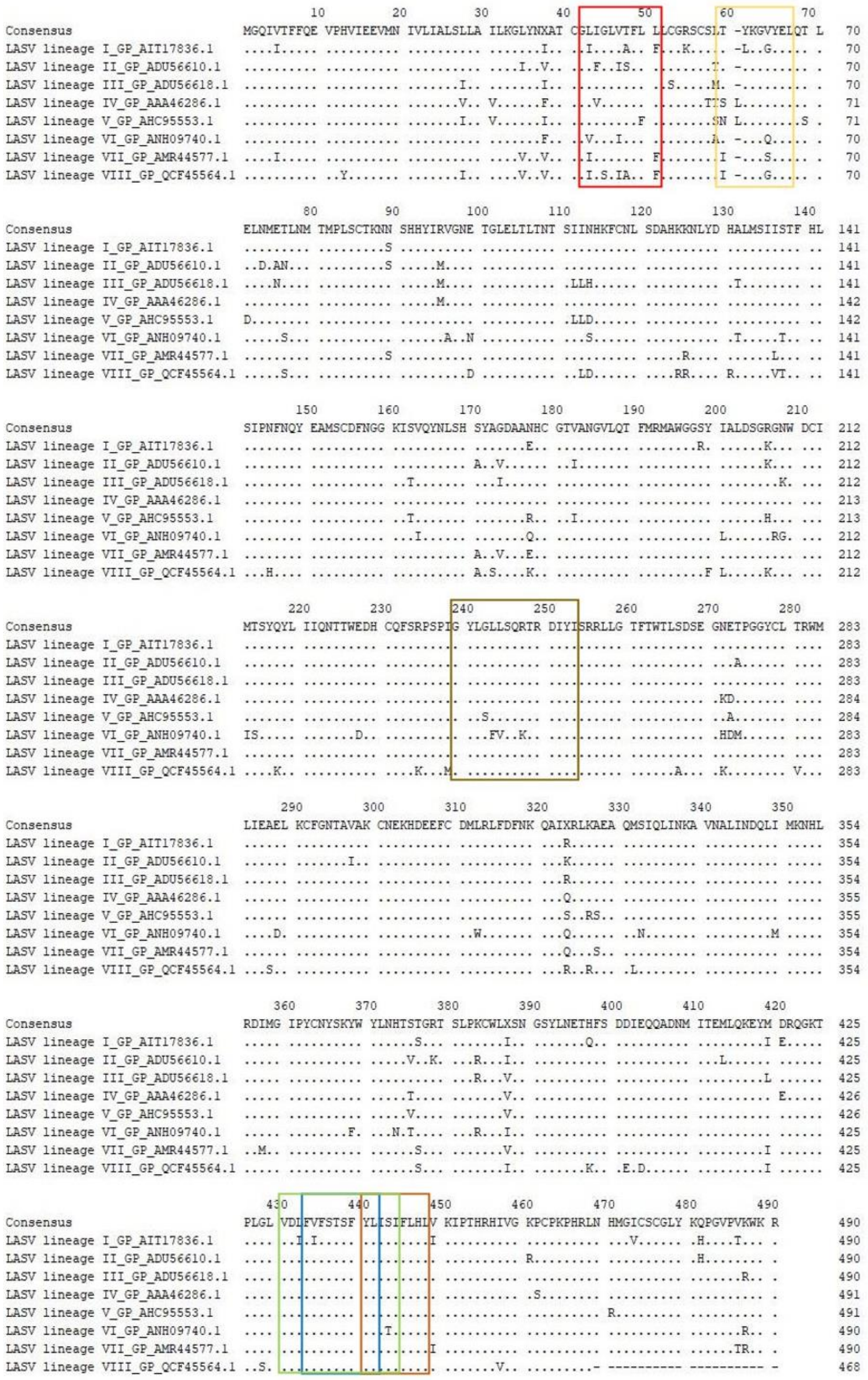

Supplementary Figure S4: Alignment of amino acid sequences from GP from LASV lineages I-VIII. LASV lineages/prototype strain sources: Lineage I- Pinneo-NIG-1969: Genbank Accession Number: AIT17836.1, Lineage II- Nig08-04: Genbank Accession Number: ADU56610.1, Lineage III- Nig08-A19: Genbank Accession Number: ADU56618.1, Lineage IV- Josiah strain: Genbank Accession Number: AAA46286.1, Lineage V-Soromba: Genbank Accession Number: AHC95553.1, Lineage VI-KAK-428: Genbank Accession Number: ANH09740.1; Lineage VII- Togo strain: Genbank Accession Number: AMR44577.1; Lineage VIII-Odo-akaba 13: Genbank Accession Number: QCF45564.1. The colored squares highlight the tested epitope regions G_{42-51} (red), G_{60-68} (yellow), $G_{434-442}$ (blue), $G_{441-449}$ (brown), $G_{240-254}$ (olive), and $G_{431-445}$ (green). Analysis was done using Geneious Prime Version 2025.0.2.

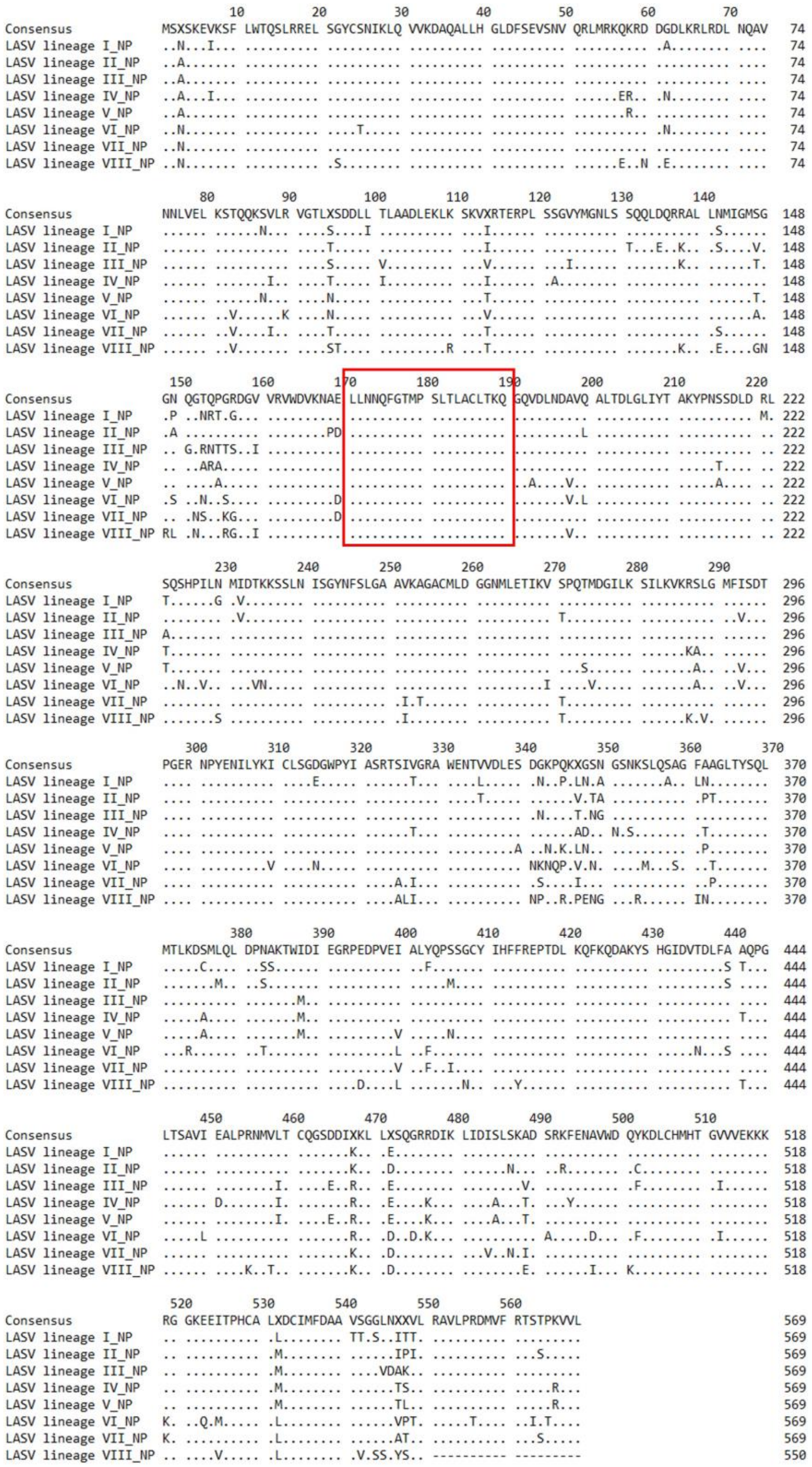

Supplementary Figure S5: Alignment of amino acid sequences from NP from LASV lineages I-VIII. LASV strains Josiah (lineage IV) and Togo (lineage VII) differ in a total of 55 amino acids. LASV lineages/prototype strain sources: Lineage I- Pinneo-NIG-1969: Genbank Accession Number: AIT17837.1, Lineage II- Nig08-04: Genbank Accession Number: ADU56611.1, Lineage III- Nig08-A19: Genbank Accession Number: ADU56619.1, Lineage IV- Josiah strain: Genbank Accession Number: AAA46285.1, Lineage V-Soromba: Genbank Accession Number: AHC95552.1, Lineage VI-KAK-428: Genbank Accession Number: ANH09741.1; Lineage VII-Togo strain: Genbank Accession Number: AMR44578.1; Lineage VIII-Odo-akaba 13: Genbank Accession Number: QCF45565.1. The red square highlights the epitope region $N_{171-190}$, which is conserved in all eight LASV lineages. Analysis was done using Geneious Prime Version 2025.0.2.

Supplementary Table S1: Predicted/published LASV-NP-, LASV-GP- and VACV-specific peptides used for ELISPOT and ICS-FACS

Name	Sequence	Pool	MHC Allele	Length (aa)	Reference
$A6L_{6-14}$	VLYDEFVTI	-	HLA*02:01	9	[1]
N_{38-47}	LLHGLDFSEV	NP1	HLA*02:01	10	-
$N_{530-539}$	ALLDCIMFDA	-	HLA*02:01	10	[2, 3], partially identical
$N_{480-489}$	KLIDVSLNKI	-	HLA*02:01	10	-
$N_{364-373}$	GLTYSQLMTL	NP1	HLA*02:01	10	[2, 3], partially identical
$N_{174-188}$	NQFGTMPSLTLACLT	-	HLA-DRB1*01:01	15	[4]
$N_{242-256}$	SGYNFSLGAAIKTGA	-	HLA-DRB1*01:01	15	[3], partially identical
$N_{176-190}$	FGTMPSLTLACLTKQ	-	HLA-DRB1*01:01	15	[4]
G_{42-51}	GLVGLVTFLL	-	HLA*02:01	10	[2, 3]
G_{60-68}	SLYKGVYEL	-	HLA*02:01	9	[2, 5]
$G_{434-442}$	FVFSTSFYL	GP1	HLA*02:01	9	-
$G_{441-449}$	YLISIFLHL	GP1	HLA*02:01	9	[2, 3]
$G_{240-254}$	GYLGLLSQRTRDIYI	GP2	HLA-DRB1*01:01	15	-
$G_{431-445}$	VDLFVFSTSFYLISI	GP2	HLA-DRB1*01:01	15	[3], partially identical

Supplementary Table S2: Overlapping LASV-NP peptides (15mer) used for ELISPOT and ICS-FACS.

Name	Sequence	Pool	Name	Sequence	Pool
N1	MSNSKEVKSFLWTQS	V1, H1	N41	VRVWDVKNADLLNNQ	V8, H4
N2	KEVKSFLWTQSLRRE	V2, H1	N42	DVKNADLLNNQFGTM	V9, H4
N3	SFLWTQSLRRELSGY	V3, H1	N43	ADLLNNQFGTMPSLT	V10, H4
N4	TQSLRRELSGYCSNI	V4, H1	N44	NNQFGTMPSLTLACL	V11, H4
N5	RRELSGYCSNIKLQV	V5, H1	N45	GTMPSLTLACLTKQG	V1, H5
N6	SGYCSNIKLQVVKDA	V6, H1	N46	SLTLACLTKQGQVDL	V2, H5
N7	SNIKLQVVKDAQALL	V7, H1	N47	ACLTKQGQVDLNDAV	V3, H5
N8	LQVVKDAQALLHGLD	V8, H1	N48	KQGQVDLNDAVQALT	V4, H5
N9	KDAQALLHGLDFSEV	V9, H1	N49	VDLNDAVQALTDLGL	V5, H5
N10	ALLHGLDFSEVSNVQ	V10, H1	N50	DAVQALTDLGLIYTA	V6, H5
N11	GLDFSEVSNVQRLMR	V11, H1	N51	ALTDLGLIYTAKYPN	V7, H5
N12	SEVSNVQRLMRKQKR	V1, H2	N52	LGLIYTAKYPNSSDL	V8, H5
N13	NVQRLMRKQKRDDGD	V2, H2	N53	YTAKYPNSSDLDRLS	V9, H5
N14	LMRKQKRDDGDLKRL	V3, H2, P1	N54	YPNSSDLDRLSQSHP	V10, H5
N15	QKRDDGDLKRLRDLN	V4, H2, P1	N55	SDLDRLSQSHPILNM	V11, H5
N16	DGDLKRLRDLNQAVN	V5, H2	N56	RLSQSHPILNMIDTK	V1, H6
N17	KRLRDLNQAVNNLVE	V6, H2	N57	SHPILNMIDTKKSSL	V2, H6
N18	DLNQAVNNLVELKSV	V7, H2	N58	LNMIDTKKSSLNISG	V3, H6, P2
N19	AVNNLVELKSVQQKS	V8, H2	N59	DTKKSSLNISGYNFS	V4, H6, P2
N20	LVELKSVQQKSILRV	V9, H2	N60	SSLNISGYNFSLGAA	V5, H6
N21	KSVQQKSILRVGTLT	V10, H2	N61	ISGYNFSLGAAIKTG	V6, H6
N22	QKSILRVGTLTSDDL	V11, H2	N62	NFSLGAAIKTGACML	V7, H6
N23	LRVGTLTSDDLLTLA	V1, H3	N63	GAAIKTGACMLDGGN	V8, H6
N24	TLTSDDLLTLAADLE	V2, H3	N64	KTGACMLDGGNMLET	V9, H6
N25	DDLLTLAADLEKLKS	V3, H3	N65	CMLDGGNMLETIKVT	V10, H6
N26	TLAADLEKLKSKVTR	V4, H3	N66	GGNMLETIKVTPQTM	V11, H6
N27	DLEKLKSKVTRTERP	V5, H3	N67	LETIKVTPQTMDGIL	V1, H7
N28	LKSKVTRTERPLSSG	V6, H3	N68	KVTPQTMDGILKSIL	V2, H7
N29	VTRTERPLSSGVYMG	V7, H3	N69	QTMDGILKSILKVKR	V3, H7
N30	ERPLSSGVYMGNLSS	V8, H3	N70	GILKSILKVKRSLGM	V4, H7
N31	SSGVYMGNLSSQQLD	V9, H3	N71	SILKVKRSLGMFISD	V5, H7
N32	YMGNLSSQQLDQRRA	V10, H3	N72	VKRSLGMFISDTPGE	V6, H7
N33	LSSQQLDQRRALLSM	V11, H3	N73	LGMFISDTPGERNPY	V7, H7
N34	QLDQRRALLSMIGMS	V1, H4	N74	ISDTPGERNPYENIL	V8, H7
N35	RRALLSMIGMSGGNQ	V2, H4	N75	PGERNPYENILYKIC	V9, H7
N36	LSMIGMSGGNQNSQP	V3, H4	N76	NPYENILYKICLSGD	V10, H7
N37	GMSGGNQNSQPKGDG	V4, H4	N77	NILYKICLSGDGWPY	V11, H7
N38	GNQNSQPKGDGVVRV	V5, H4	N78	KICLSGDGWPYIASR	V1, H8
N39	SQPKGDGVVRVWDVK	V6, H4	N79	SGDGWPYIASRTAII	V2, H8
N40	GDGVVRVWDVKNADL	V7, H4	N80	WPYIASRTAIIGRAW	V3, H8, P3

N81	ASRTAIIGRAWENTV	V4, H8, P3
N82	AIIGRAWENTVVDLE	V5, H8
N83	RAWENTVVDLESDSK	V6, H8
N84	NTVVDLESDSKPQKI	V7, H8
N85	DLESDSKPQKIGSNG	V8, H8
N86	DSKPQKIGSNGSNKS	V9, H8
N87	QKIGSNGSNKSLQSA	V10, H8
N88	SNGSNKSLQSAGFAP	V11, H8
N89	NKSLQSAGFAPGLTY	V1, H9
N90	QSAGFAPGLTYSQLM	V2, H9
N91	FAPGLTYSQLMTLKD	V3, H9
N92	LTYSQLMTLKDSMLQ	V4, H9
N93	QLMTLKDSMLQLDPN	V5, H9
N94	LKDSMLQLDPNAKTW	V6, H9
N95	MLQLDPNAKTWIDIE	V7, H9
N96	DPNAKTWIDIEGRPE	V8, H9
N97	KTWIDIEGRPEDPVE	V9, H9
N98	DIEGRPEDPVEVALF	V10, H9
N99	RPEDPVEVALFQPIS	V11, H9
N100	PVEVALFQPISGCYI	V1, H10
N101	ALFQPISGCYIHFFR	V2, H10
N102	PISGCYIHFFREPTD	V3, H10
N103	CYIHFFREPTDLKQF	V4, H10
N104	FFREPTDLKQFKQDA	V5, H10
N105	PTDLKQFKQDAKYSH	V6, H10
N106	KQFKQDAKYSHGIDV	V7, H10
N107	QDAKYSHGIDVTDLF	V8, H10
N108	YSHGIDVTDLFAAQP	V9, H10
N109	IDVTDLFAAQPGLTS	V10, H10
N110	DLFAAQPGLTSAVIE	V11, H10
N111	AQPGLTSAVIEALPR	V1, H11
N112	LTSAVIEALPRNMVL	V2, H11
N113	VIEALPRNMVLTCQG	V3, H11
N114	LPRNMVLTCQGSDDI	V4, H11
N115	MVLTCQGSDDIKKLL	V5, H11
N116	CQGSDDIKKLLDSQG	V6, H11
N117	DDIKKLLDSQGRRDI	V7, H11
N118	KLLDSQGRRDIKLID	V8, H11
N119	SQGRRDIKLIDVSLN	V9, H11
N120	RDIKLIDVSLNKIDS	V10, H11
N121	LIDVSLNKIDSRKFE	V11, H11
N122	SLNKIDSRKFENAVW	V1, H1
N123	IDSRKFENAVWDQYK	V2, H1
N124	KFENAVWDQYKDLCH	V3, H1
N125	AVWDQYKDLCHMHTG	V4, H1
N126	QYKDLCHMHTGVVVE	V5, H1
N127	LCHMHTGVVVEKKKK	V6, H1
N128	HTGVVVEKKKKGGKE	V7, H1
N129	VVEKKKKGGKEEITP	V8, H1
N130	KKKGGKEEITPHCAL	V9, H1
N131	GKEEITPHCALLDCI	V10, H1
N132	ITPHCALLDCIMFDA	V11, H1
N133	CALLDCIMFDAAVSG	V1, H2
N134	DCIMFDAAVSGGLNA	V2, H2
N135	FDAAVSGGLNATVLR	V3, H2, P4
N136	VSGGLNATVLRAVLP	V4, H2, P4
N137	LNATVLRAVLPRDMV	V5, H2
N138	VLRAVLPRDMVFRTS	V6, H2
N139	VLPRDMVFRTSSPKV	V7, H2
N140	DMVFRTSSPKVVL	V8, H2

Supplementary Table S3: Antibodies for intracellular cytokine staining

Antibody	Manufacturer	Catalogue No.	Dilution
Anti-mouse CD3 PE/Cy7	Biolegend	100220	1:100
Anti-mouse CD4 Brilliant Violet 421™	Biolegend	100437	1:600
Anti-mouse CD8α Alexa Fluor® 488	Biolegend	100723	1:300
TruStain FcX™ (anti-mouse CD16/32)	Biolegend	101320	1:500
Anti-mouse IFN-γ APC	Biolegend	505810	1:300
Anti-mouse TNF-α PE	Biolegend	506306	1:300

Supplementary Table S4: GP amino acid percent identity matrix among major (I-IV) and proposed (V-VIII) LASV lineages. LASV lineages/prototype strain sources: Lineage I- Pinneo-NIG-1969: Genbank Accession Number: AIT17836.1, Lineage II- Nig08-04: Genbank Accession Number: ADU56610.1, Lineage III- Nig08-A19: Genbank Accession Number: ADU56618.1, Lineage IV- Josiah strain: Genbank Accession Number: AAA46286.1, Lineage V-Soromba: Genbank Accession Number: AHC95553.1, Lineage VI-KAK-428: Genbank Accession Number: ANH09740.1; Lineage VII- Togo strain: Genbank Accession Number: AMR44577.1; Lineage VIII-Odo-akaba 13: Genbank Accession Number: QCF45564.1. Analysis was done using Geneious Prime Version 2025.0.2.

	**Lineage I**	**Lineage II**	**Lineage III**	**Lineage IV**	**Lineage V**	**Lineage VI**	**Lineage VII**	**Lineage VIII**
**Lineage I**	100%	92.2%	92.7%	92.9%	91.4%	89.0%	95.1%	90.4%
**Lineage II**	92.2%	100%	93.3%	93.5%	91.9%	89.8%	92.7%	88.2%
**Lineage III**	92.7%	93.3%	100%	94.7%	94.5%	91.2%	93.5%	88.9%
**Lineage IV**	92.9%	93.5%	94.7%	100%	94.5%	91.4%	93.5%	88.5%
**Lineage V**	91.4%	91.9%	94.5%	94.5%	100%	89.2%	92.3%	88.5%
**Lineage VI**	89.0%	89.8%	91.2%	91.4%	89.2%	100%	89.8%	86.5%
**Lineage VII**	95.1%	92.7%	93.5%	93.5%	92.3%	89.8%	100%	91.0%
**Lineage VIII**	90.4%	88.2%	88.9%	88.5%	88.5%	86.5%	91.0%	100%

Supplementary Table S5: NP amino acid percent identity matrix among major (I-IV) and proposed (V-VIII) LASV lineages. LASV lineages/prototype strain sources: Lineage I- Pinneo-NIG-1969: Genbank Accession Number: AIT17837.1, Lineage II- Nig08-04: Genbank Accession Number: ADU56611.1, Lineage III- Nig08-A19: Genbank Accession Number: ADU56619.1, Lineage IV- Josiah strain: Genbank Accession Number: AAA46285.1, Lineage V-Soromba: Genbank Accession Number: AHC95552.1, Lineage VI-KAK-428: Genbank Accession Number: ANH09741.1; Lineage VII- Togo strain: Genbank Accession Number: AMR44578.1; Lineage VIII-Odo-akaba 13: Genbank Accession Number: QCF45565.1. Analysis was done using Geneious Prime Version 2025.0.2.

	**Lineage I**	**Lineage II**	**Lineage III**	**Lineage IV**	**Lineage V**	**Lineage VI**	**Lineage VII**	**Lineage VIII**
**Lineage I**	100%	90.0%	90.3%	90.5%	90.0%	86.6%	90.7%	87.5%
**Lineage II**	90.0%	100%	91.2%	89.6%	90.7%	88.4%	92.4%	87.1%
**Lineage III**	90.3%	91.2%	100%	91.7%	92.4%	87.7%	91.4%	88.0%
**Lineage IV**	90.5%	89.6%	91.7%	100%	94.0%	85.9%	90.3%	86.5%
**Lineage V**	90.0%	90.7%	92.4%	94.0%	100%	87.7%	90.7%	86.5%
**Lineage VI**	86.6%	88.4%	87.7%	85.9%	87.7%	100%	88.4%	84.7%
**Lineage VII**	90.7%	92.4%	91.4%	90.3%	90.7%	88.4%	100%	89.8%
**Lineage VIII**	87.5%	87.1%	88.0%	86.5%	86.5%	84.7%	89.8%	100%

References

1. **Pasquetto V, Bui HH, Giannino R, Banh C, Mirza F et al.** HLA-A*0201, HLA-A*1101, and HLA-B*0702 transgenic mice recognize numerous poxvirus determinants from a wide variety of viral gene products. *J Immunol* 2005;175(8):5504-5515.
2. **Botten J, Alexander J Fau - Pasquetto V, Pasquetto V Fau - Sidney J, Sidney J Fau - Barrowman P, Barrowman P Fau - Ting J et al.** Identification of protective Lassa virus epitopes that are restricted by HLA-A2. (0022-538X (Print)).
3. **Boesen A, Sundar K, Coico R**. Lassa fever virus peptides predicted by computational analysis induce epitope-specific cytotoxic-T-lymphocyte responses in HLA-A2.1 transgenic mice. *Clin Diagn Lab Immunol* 2005;12(10):1223-1230.
4. **ter Meulen J, Badusche M, Kuhnt K, Doetze A, Satoguina J et al.** Characterization of human CD4(+) T-cell clones recognizing conserved and variable epitopes of the Lassa virus nucleoprotein. *J Virol* 2000;74(5):2186-2192.
5. **Sakabe S, Hartnett JN, Ngo N, Goba A, Momoh M et al.** Identification of Common CD8(+) T Cell Epitopes from Lassa Fever Survivors in Nigeria and Sierra Leone. *J Virol* 2020;94(12).
